# Charge-engineered cellulose membranes from wood waste for ionic thermoelectric energy conversion

**DOI:** 10.1039/d6ra03519a

**Published:** 2026-08-05

**Authors:** Anjali Ashokan, Rupa Ranjani Palanisamy, Kafil M. Razeeb, Subhajit Biswas, Justin D. Holmes

**Affiliations:** a School of Chemistry, University College Cork Cork T12 YN60 Ireland s.biswas@ucc.ie j.holmes@ucc.ie; b AMBER Centre, Sustainability Institute, University College Cork Cork T23 XE10 Ireland; c Micro-Nano System Centre, Tyndall National Institute Cork T12 R5CP Ireland

## Abstract

Approximately 60–67% of global energy production is lost as low-grade heat. Recovering even a small fraction of this waste heat could materially improve overall energy efficiency. Here, we present a sustainable ionic thermoelectric platform based on cellulose membranes derived from waste wood chips and surface-functionalised with *N*-[(3-trimethoxysilyl)propyl]ethylenediamine triacetic acid trisodium salt (TMSDA). Silanisation introduces tricarboxylate groups, increasing the carboxylate content from 0.16 to 0.86 mmol g^−1^ (∼5.4×) and enhancing the zeta potential to −33.5 mV. Fitting the concentration-dependent ionic conductivity data across a wide range of KCl concentrations revealed a nearly 9-fold increase in surface conductivity and a crossover concentration of c* ≈1.7 × 10^−3^ M, supporting surface-governed ion transport in the dilute regime. At 10^−4^ M KCl, TMSDA-cellulose membranes exhibited an ionic conductivity of 1.51 mS cm^−1^, ∼13× higher than pristine cellulose, driven by enhanced counter-ion mobility within charged porous pathways. When integrated into a stacked thermocell and subjected to a 10 K temperature gradient, the membranes generated an open-circuit thermovoltage (*V*_oc_) of −128 mV, corresponding to an apparent ionic Seebeck coefficient (*S*_i_ = *V*_oc_/Δ*T*) of −12.8 mV K^−1^ (for Δ*T* = 10 K) and a power factor of 24.7 ± 6.8 µW m^−1^ K^−2^. These values represent an approximately 3.2-fold improvement over pristine membranes and rank among the highest reported for cellulose-based ionic thermoelectrics operating in dilute, neutral aqueous electrolytes. Unlike many state-of-the-art systems that require ionic liquids or harsh electrolytes, our approach combines biomass valorisation, scalable membrane assembly, and green functionalisation chemistry to establish charge-engineered, waste-derived cellulose as a versatile and environmentally responsible platform for ionic thermoelectrics.

## Abstract 2

Approximately 60–67% of global energy production is lost as low-grade heat.

## Graphical abstract

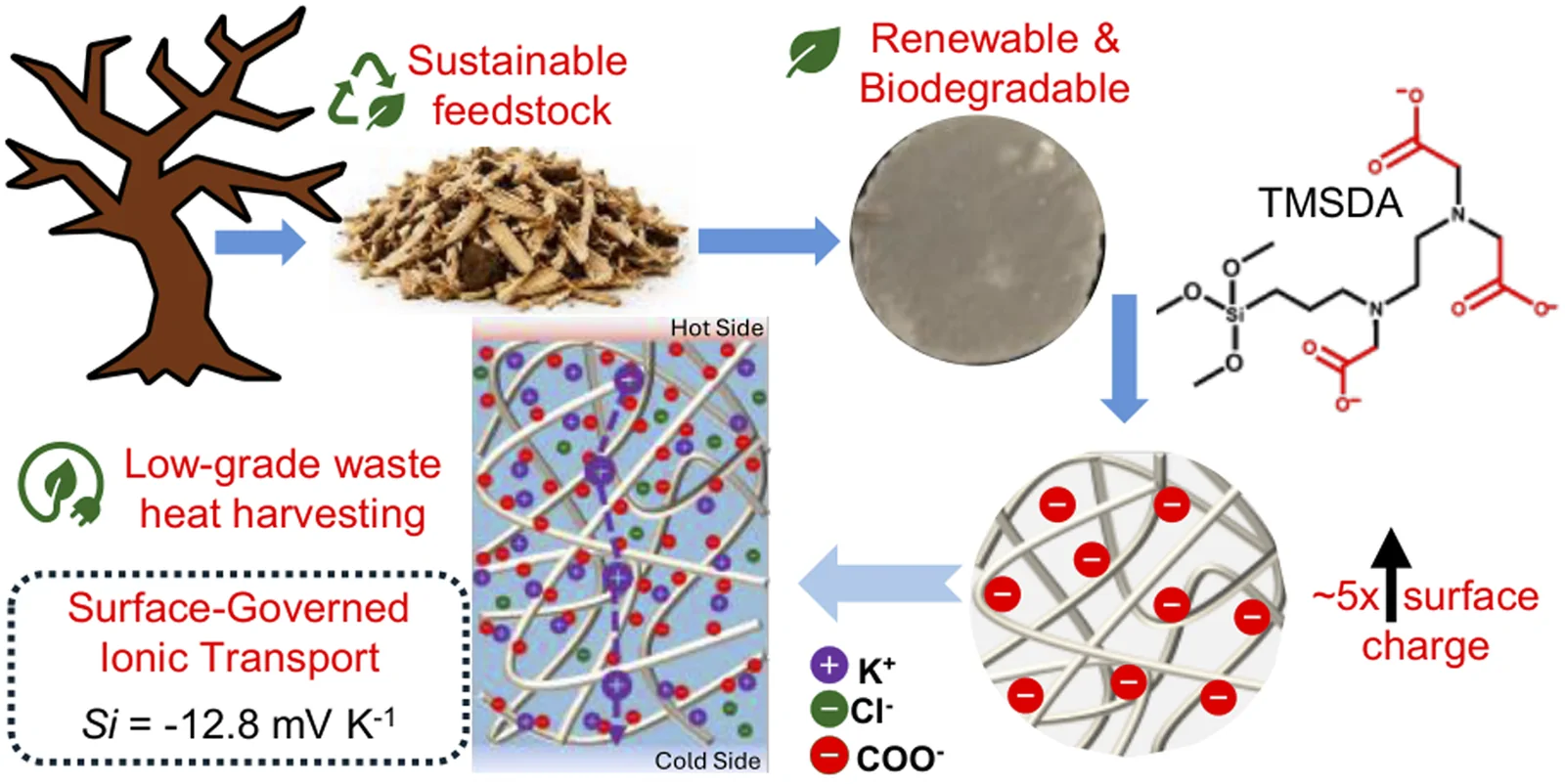

## Introduction

Meeting global energy demands sustainably remains one of the most pressing scientific and technological challenges. A significant portion of generated energy is dissipated as waste heat, much of it at low temperature, representing an underexploited renewable resource for electricity generation.^1,2^ Converting even a fraction of this waste heat into electricity would help improve overall energy efficiency and reduce associated emissions. Solid-state thermoelectrics, based on the Seebeck effect,^3^ offer a direct means of converting heat into electricity. However, their practical application is hindered by low output under modest temperature gradients, high cost, and reliance on scarce or toxic elements.^4^ These drawbacks have motivated growing interest in scalable, sustainable alternatives.

Nanofluidic systems have recently emerged as promising platforms for ionic thermoelectrics.^5–8^ Unlike conventional thermoelectrics, which rely on electronic charge carriers, ionic thermoelectrics exploit the thermally driven diffusion of ions (the Soret effect) to generate a thermovoltage under a temperature gradient.^9^ When an electrolyte-infiltrated nanofluidic membrane is exposed to such a gradient, cations and anions migrate at different rates between the hot and cold sides.^10^ In charge-selective membranes, where surface charges are introduced through functionalisation, this mobility asymmetry leads to net charge separation and the development of an internal electric field, which manifests as a measurable thermovoltage.^5,9,11^ Therefore, the strength of this response depends strongly on the density of fixed interfacial charges: higher interfacial charge promotes counter-ion accumulation, excludes co-ions, and improves ionic selectivity, thereby enhancing thermoelectric performance.

Recent studies have shown that incorporating membranes can substantially enhance thermally induced voltage generation.^10,12,13^ For example, Van Toan *et al.*^10^ demonstrated a thermoelectric power battery using an Al_2_O_3_ nanochannel membrane, which increased the ionic Seebeck coefficient (*S*_i_) of KCl electrolyte from 0.03 to 1.8 mV K^−1^, leading to ≈ 5900% increase with nanochannels. Likewise, Sultana *et al.*^14^ reported that a polarised electrospun membrane, inspired by thermoresponsive ion channels in human skin, boosted the ionic Seebeck coefficient from 5 ± 1.3 to ∼15 mV K^−1^ by introducing a ferroelectric polymer fibre matrix at the cold electrode. These advances highlight the role of membrane design in improving ionic thermoelectric performance and point toward the potential of soft, flexible, and biodegradable membranes as a new class of sustainable ionic thermoelectrics.

In this context, cellulose has emerged as a desirable candidate. Its abundance, biodegradability, widespread availability, hierarchical fibrous structure, and hydroxyl-rich backbone make it an excellent platform for surface functionalisation and nanofluidic device design.^15–17^ Additionally, cellulose's inherently low thermal conductivity helps maintain temperature gradients across the membrane, a desirable property for thermoelectric performance. While native cellulose carries only a modest negative surface charge arising from weakly dissociable hydroxyl groups,^17^ the high density of these functional groups enables controlled chemical modification to introduce fixed charges and enhance ionic selectivity.^18^ Importantly, sourcing cellulose from industrial or agricultural waste streams enhances circular-economy principles in membrane fabrication, supporting sustainable nanofluidic device platforms without depending on synthetic polymers.

A variety of functionalisation strategies, including silane coupling, TEMPO oxidation, sulfonation, and carboxymethylation, have been reported to increase the surface charge of cellulose.^19–22^ Such modifications fundamentally alter interfacial ion transport by increasing the density of fixed ionisable groups or charged sites, strengthening electrostatic interactions with counter-ions, and suppressing co-ion migration within confined transport pathways. As a result, ion conduction becomes increasingly surface-governed, particularly under dilute electrolyte conditions: the higher the surface charge, the higher the ionic mobility. For instance, Li *et al.*^17^ demonstrated that TEMPO oxidation of cellulose nanofibres, which converts hydroxyl groups into ionisable carboxyl groups, led to a 1.8-fold increase in nanofluidic conductivity in KCl relative to pristine cellulose, highlighting the critical role of fixed ionisable group content and interfacial charge in governing interfacial ion mobility. Under thermal gradients, such charge-induced selectivity is especially critical, as enhanced counter-ion enrichment amplifies thermodiffusive asymmetry between the species (co-ions and counter-ions) and increases the thermovoltage output.^16^ Chen *et al.*^23^ reported a TEMPO-oxidised, carboxylated bacterial cellulose double-network hydrogel that, when doped with lithium bis(trifluoromethanesulfonyl)imide (LiTFSI), delivered an ionic Seebeck coefficient of 11.53 mV K^−1^ under a 20 K temperature gradient, further emphasising the significance of interfacial charge control in determining thermoelectric response. Despite these advances, many high-performance cellulose-based systems depend on polymer electrolytes, ionic liquids, or strongly acidic or alkaline conditions, raising concerns about sustainability and stability. Moreover, hydrogel-based designs, though effective for ion transport, often lack mechanical robustness and face scalability limitations. Therefore, the main challenge is to develop rational surface-charge engineering strategies that achieve strong interfacial ion selectivity while functioning in benign, neutral electrolytes.

Although cellulose-based ionic thermoelectric materials have attracted growing interest, many reported systems depend on TEMPO oxidation, charged polymer additives, hydrogels, ionic liquids, or concentrated acidic/alkaline electrolytes to achieve high thermoelectric performance. Moreover, relatively few studies have quantitatively connected fixed ionisable group content with surface-governed ion transport and its impact on ionic thermoelectric behaviour. In this work, waste-derived cellulose membranes are functionalised with a tricarboxylate silane, TMSDA, to introduce a high density of fixed carboxylate groups within electrolyte-filled porous pathways, promoting interfacial ion transport under neutral aqueous conditions. By combining concentration-dependent conductivity analysis with ionic thermoelectric measurements, we extract the apparent surface conductivity, *κ*_surf_, and the crossover concentration, c*, thereby linking TMSDA-induced charge engineering to surface-dominated ion transport. Operating the TMSDA-cellulose membranes below c* in dilute KCl yields a high apparent ionic Seebeck coefficient of −12.8 mV K^−1^ and an enhanced power factor in stacked devices. Because the membranes are derived from industrial wood waste and processed using aqueous chemistry and a neutral KCl electrolyte, this approach combines biomass valorisation, green functionalisation, and mechanistic charge–transport design in a sustainable cellulose platform.

## Experimental

### Materials

Wood chips were sourced from Medite Smartply (Clonmel, Ireland). Sodium hydroxide (NaOH), hydrochloric acid (HCl), potassium chloride (KCl), sodium sulfite (Na_2_SO_3_), hydrogen peroxide (H_2_O_2_, 35 wt% solution), ethanol, and SYLGARD-184 (curing agent) were obtained from Sigma-Aldrich and used as received without further purification. *N*-[(3-trimethoxysilyl)propyl]ethylenediamine triacetic acid trisodium salt (TMSDA, 35% in water) was purchased from Dough Discovery – fluorochem and used as received. Deionised water was used throughout all experiments.

### Synthesis and functionalisation

#### Preparation of cellulose membranes

Cellulose was extracted from wood chips following a reported method.^16,17^ Briefly, wood chips were boiled for 10 h in 100 mL of an aqueous solution containing 20 g of sodium hydroxide (NaOH) and 5 g of sodium sulfite (Na_2_SO_3_) to remove lignin and release cellulose fibres. The resulting pulp was repeatedly boiled in water (three cycles) to remove residual chemicals, then bleached in 50 mL of 30 wt% hydrogen peroxide (H_2_O_2_) until a white colouration indicated successful delignification (see Fig. S1 in SI). The cellulose was thoroughly rinsed with deionised water to remove residual peroxide, then dried and stored for further use. The extracted cellulose was dispersed at a concentration of 15 mg mL^−1^ in water under magnetic stirring. A 20 mL aliquot of this dispersion was vacuum-filtered through a 9.6 cm^2^ filtration setup to form membranes, which were subsequently dried and easily peeled from the filter as free-standing white films.

#### Synthesis of TMSDA-functionalised cellulose

To increase the fixed ionisable group content and strengthen the negative interfacial charge, cellulose membranes were functionalised with TMSDA. This tricarboxylate-bearing silane reacts with cellulose hydroxyl groups (see Scheme S1 in SI). A modified silanisation protocol, adapted from ethanol–water-based methods with thermal curing and post-reaction washing, was employed.^24,25^ Briefly, 1 g of cellulose membrane was immersed in 30 mL of a 1 : 1 ethanol–water mixture containing 10 wt% TMSDA. The mixture was degassed under vacuum for 1 h to remove air bubbles and promote uniform grafting, then left overnight in the reaction medium, and finally cured at 60 °C for an additional 12 h. The membranes were thoroughly washed with ethanol–water to remove unreacted species, dried, and stored for further characterisation.

#### Synthesis of TEMPO-oxidised cellulose

TEMPO-mediated oxidation of cellulose was performed following a previously reported protocol.^26^ Briefly, for 1 g of cellulose membrane in 100 mL of deionised water, 2,2,6,6-tetramethylpiperidine-1-oxyl (TEMPO, 0.016 g) and sodium bromide (NaBr, 0.1 g) were added to the suspension. The reaction was initiated by dropwise addition of a sodium hypochlorite solution (NaClO, 5 mmol per g of cellulose) while maintaining the pH at 10–10.5 through continuous addition of 0.5 M sodium hydroxide (NaOH). The reaction was carried out at room temperature for 2.5 h. Upon completion, the reaction was quenched by adjusting the pH to 7 with HCl. The oxidised cellulose was thoroughly washed with 1 M HCl to remove residual reagents, then solvent-exchanged with acetone and toluene.

### Characterisation

#### Structural and surface characterisation

The chemical composition and surface properties of the cellulose-based membranes were examined using a range of techniques. Fourier-transform infrared (FTIR) spectroscopy (PerkinElmer) was performed on 2 × 2 cm membrane samples. Wettability was evaluated on identical samples by measuring water contact angles with an Ossila contact angle instrument. Thermal stability was assessed by thermogravimetric analysis (TGA), heating samples from room temperature to 600 °C at 20 °C min^−1^ under nitrogen. Surface morphology was observed using scanning electron microscopy (SEM, Quanta 650) on ∼0.3 × 0.3 cm sections, which were gold-coated for 30 s under vacuum. Zeta potential was measured using a Malvern Zetasizer Nano S90 by dispersing 0.01 wt% pristine or TMSDA-functionalised cellulose in deionised water and stirring overnight.

#### Conductometric titration

The concentration of negatively charged functional groups was determined by conductometric titration, based on the volume of strong base required to neutralise the weak acidic groups associated with the cellulose membrane.^16^ Since each-carboxylic acid group (COOH) contributes one negative charge upon deprotonation, the measured acid content provides a mass-normalised fixed ionisable group content (*Γ*_COOH_). Pristine and TMSDA-functionalised cellulose membranes (40 mg) were dispersed in 40 mL of deionised water under continuous stirring. To fully protonate carboxyl groups, 1 M HCl was added until the pH fell below 4, followed by the addition of NaCl. The dispersion was then titrated with 0.01 M NaOH while conductivity was recorded in real time using an Orion Versa Star Pro conductivity meter (Thermo Fisher Scientific). The titration-derived fixed ionisable group content, *Γ*_COOH_, was calculated according to eqn (1):^16^1
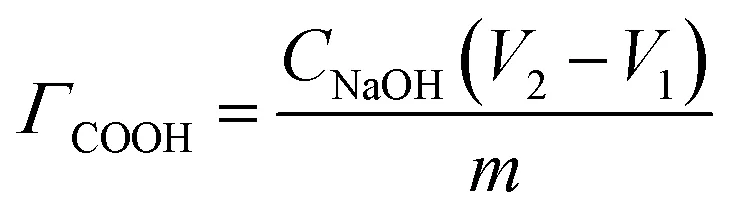
where *Γ*_COOH_ is the titration-derived fixed ionisable group content, expressed in mmol g^−1^. In this form of the equation, *C*_NaOH_ is given in mol L^−1^, (*V*_1_) and (*V*_2_) are given in mL, and (*m*) is given in g. *C*_NaOH_ is the concentration of the NaOH titrant, (*m*) is the dry mass of the cellulose sample, (*V*_1_) is the volume of NaOH required to neutralise the added strong acid, and (*V*_2_) is the second equivalence point. The difference (*V*_2_–*V*_1_) therefore represents the volume of NaOH required to neutralise weak acidic groups associated with the cellulose membrane.

#### Ionic conductivity measurements

Pristine and TMSDA-functionalised cellulose membranes were cut into rectangular strips and encapsulated in SYLGARD-184 silicone elastomer (see Fig. S2 in SI). After curing, reservoirs were carved at each end of the strip to expose the membrane to electrolyte. The devices were immersed in Milli-Q water for 2 days to ensure complete hydration, then soaked in an electrolyte solution for at least 1 day before testing to promote full wetting. Electrochemical measurements were performed using AgCl electrodes, prepared by immersing Ag wires in a NaOCl solution for 2 days, followed by thorough rinsing with water. Ionic conductivity was measured *via* linear sweep voltammetry (LSV),^17,27^ using a Biologic SP-50e potentiostat, with a scan rate of 10 mV s^−1^ over a potential window of ± 0.5 V. Conductivity values were calculated according to eqn (2):^28,29^2
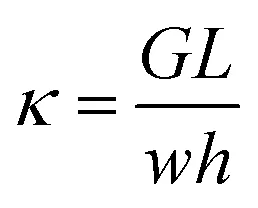
where, *κ* is the ionic conductivity (mS cm^−1^), *G* is the measured conductance (mS), *L* is the distance between electrodes (cm), *w* is the width (cm), and *h* is the thickness (cm) of the sample.

Electrochemical impedance spectroscopy (EIS) measurements were performed in the same cell geometry used for the LSV conductivity experiments, over the 10^6^–10^2^ Hz range with an AC amplitude of 10 mV (rms) using a Biologic SP-50e potentiostat. The obtained data were fitted to the equivalent circuit model, *R*_1_ + (*R*_2_‖*C*_2_), using EC-lab software. The extracted fitting parameters and additional experimental details are provided in the SI.

#### Thermoelectric characterisation using a sandwich cell setup

The thermoelectric performance of cellulose-based membranes was evaluated using a customised sandwich-cell configuration based on the modified ASTM D5470-06 standard (see Fig. S3 in SI). Membranes (1 × 1 cm), pre-infiltrated overnight with 10^−4^ M KCl, were sandwiched between two gold-coated silicon wafer electrodes and mounted between copper rods connected to a Peltier cooler (cold side) and a thermal source (hot side). An external supply powered both elements to establish a controlled temperature gradient across the membrane. Local temperatures were monitored using thermistors embedded in the copper rods, and the corresponding open-circuit voltage (*V*_oc_), *i.e.*, the potential difference between the two electrodes under zero current, was recorded using an electrochemical workstation. All measurements were performed in accordance with ASTM D5470-06.^30^ Ionic Seebeck coefficients are reported as *S*_i_ = *V*_oc_/Δ*T* from a single 10 K step and therefore represent single-point (apparent) estimates, assuming linearity over 0–10 K.

## Results and discussion

### Membrane fabrication and functionalisation

**Fig. 1:**
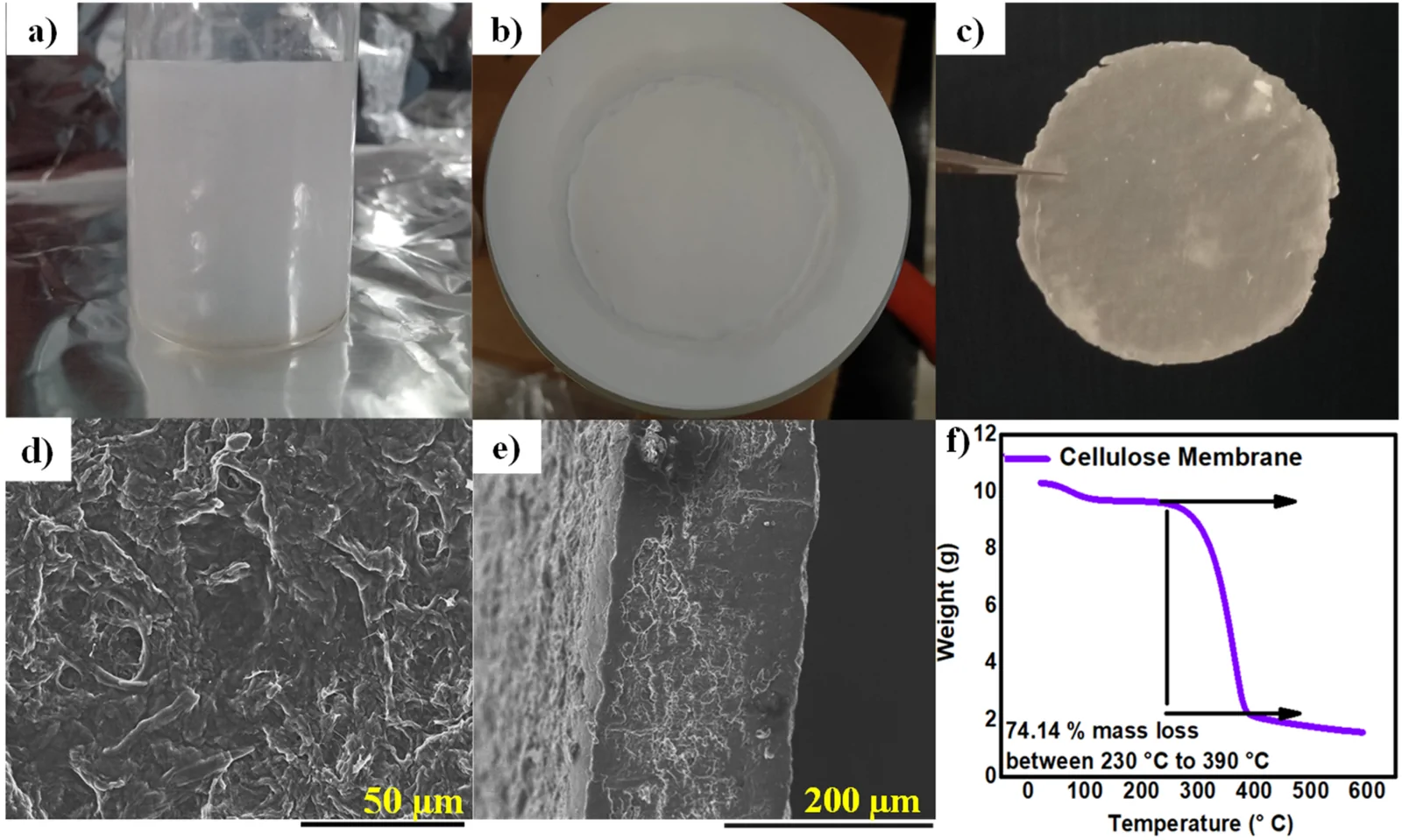
Fabrication and characterisation of cellulose membranes derived from waste wood chips: (a) cellulose dispersion in water, (b) wet membrane on the filtration support, and (c) dry free-standing membrane. SEM images of the membrane (d) surface (scale bar: 50 µm) and (e) cross-section (scale bar: 200 µm) reveal the interconnected fibrous network. (f) TGA profile of the membrane, demonstrating thermal stability within the low-grade heat region (≤100 °C).

Cellulose membranes were fabricated from waste wood chips *via* a simple sequence of alkaline pulping, bleaching, vacuum filtration and drying to yield robust, free-standing membranes (Fig. 1(a–c) and S1, see SI). Following alkaline pulping and bleaching of the waste wood chips, the extracted cellulose fibres were dispersed in water under magnetic stirring to form a homogeneous aqueous dispersion, a suspension of fibrillated cellulose fibres rather than a true solution. No cellulose dissolution or regeneration step was employed at any stage. During vacuum filtration, the fibrillated cellulose fibres assembled into a densely packed, interconnected network that was retained upon drying, producing a self-supporting membrane. The resulting structure is therefore distinct from both regenerated cellulose films prepared *via* dissolution–regeneration routes and conventional paper or filter sheets composed of coarse, loosely assembled pulp fibres. SEM imaging revealed a densely interconnected, fibrillated cellulose network (Fig. 1(d), (e) and S4, SI). Higher-magnification images reveal a porous architecture composed of overlapping fibrillated cellulose domains, with interconnected voids distributed throughout the membrane. This morphology reflects the partial disaggregation of wood fibre cell walls during alkaline pulping and bleaching, which liberates fibrillated cellulose domains that are substantially finer and more interconnected than the coarse fibres in conventional filter paper. Although the elementary fibril dimensions (∼3–4 nm) and microfibril bundles (∼10–30 nm) are below the resolution of the present SEM images, the observed porous interconnected morphology is fully consistent with literature descriptions of hierarchically fibrillated cellulose networks produced by similar alkaline pulping routes. It is corroborated by the surface-governed ion transport behaviour established in subsequent conductivity measurements.^31,32^ This architecture produces a porous fibre mesh with interconnected voids that act as continuous electrolyte-filled transport pathways, including nanoscale inter-fibrillar environments. At the same time, cross-sectional imaging confirmed a uniform fibre network distributed across the ∼180 µm thick membranes. Together, these observations show that waste-derived cellulose can be processed into mechanically stable, nanostructured membranes suitable for ion transport.

Water contact angle measurements confirmed the strongly hydrophilic nature of the wood-derived cellulose membranes, with a contact angle of 41.4 ± 1.3°, ensuring rapid wetting and complete electrolyte infiltration, a prerequisite for effective ionic conduction in nanofluidic systems. Thermogravimetric analysis showed that the membranes remained stable up to ∼230 °C, with significant mass loss occurring only between 230 and 390 °C due to decomposition of the cellulose backbone (Fig. 1(f)). This thermal stability is more than sufficient for operation under near-room-temperature conditions and moderate thermal gradients typical of low-grade heat (≤100 °C). Chemical durability was further demonstrated by immersing membranes in 1 M HCl, 1 M NaOH, and deionised water for extended periods without visible degradation (see Fig. S5 in the SI), thereby establishing the waste-wood-derived membranes as hydrophilic, thermally and chemically robust scaffolds for nanofluidic applications.

**Fig. 2:**
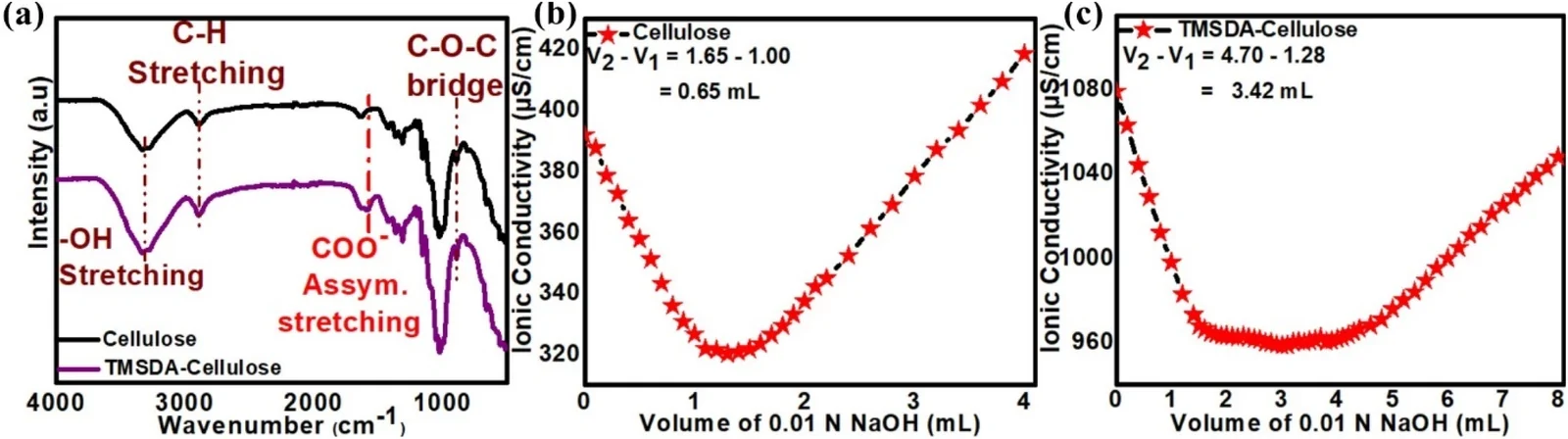
(a) FTIR spectra of pristine and TMSDA-functionalised cellulose membranes, showing characteristic cellulose absorption bands and the new carboxyl stretching peak from TMSDA. (b) Conductometric titration profile of pristine cellulose and (c) TMSDA-functionalised cellulose membranes, used to quantify surface acidic groups.

Native cellulose membranes contain abundant hydroxyl groups but only a modest density of ionisable sites, resulting in a low intrinsic surface charge and a moderately negative zeta potential. For example, a 0.01 wt% dispersion of pristine cellulose exhibited a *ζ*-potential of −21.5 ± 0.79 mV.^28,33^ To increase the fixed ionisable group content and promote ion-selective transport, the membranes were functionalised with a tricarboxylate-terminated silane (TMSDA), which is expected to graft onto cellulose *via* silanisation and condense with surface hydroxyl groups (see Scheme S1 in the SI). FTIR spectra of pristine and TMSDA-functionalised membranes (Fig. 2(a)) showed that the characteristic cellulose bands (O–H stretching ∼3300 cm^−1^, C–H stretching ∼2900 cm^−1^ and C–O–C glycosidic link ∼895 cm^−1^) were preserved,^34^ indicating that the cellulose backbone remains intact after functionalisation, while new bands associated with carboxyl group stretching were consistent with successful TMSDA functionalisation.^35^ The efficiency of surface functionalisation and the resulting increase in fixed ionisable group content were assessed using zeta potential measurements and conductometric titrations.^36,37^ Zeta potential analysis showed that the pristine cellulose membranes (−21.5 ± 0.79 mV) became significantly more negative after TMSDA functionalisation (−33.5 ± 1.66 mV), reflecting the incorporation of carboxylate groups onto the surface (see Scheme S1). Although *ζ*-potential values were obtained from 0.01 wt% dispersions, they are indicative of the surface electrostatic potential of intact membranes, since both arise from the same functionalised cellulose surfaces and ionisable carboxylate groups. Conductometric titration showed an increase in titration-derived carboxylate content from 0.16 mmol g^−1^ in pristine cellulose to 0.86 mmol g^−1^ after silanisation, corresponding to a 5.4-fold enhancement in fixed ionisable group content (see Fig. 2(b) and (c)). These results demonstrate that the mild aqueous silanisation process preserves the cellulose framework while substantially increasing the density of ionisable surface groups, producing charge-engineered cellulose membranes suitable for nanofluidic ion transport.

**Fig. 3:**
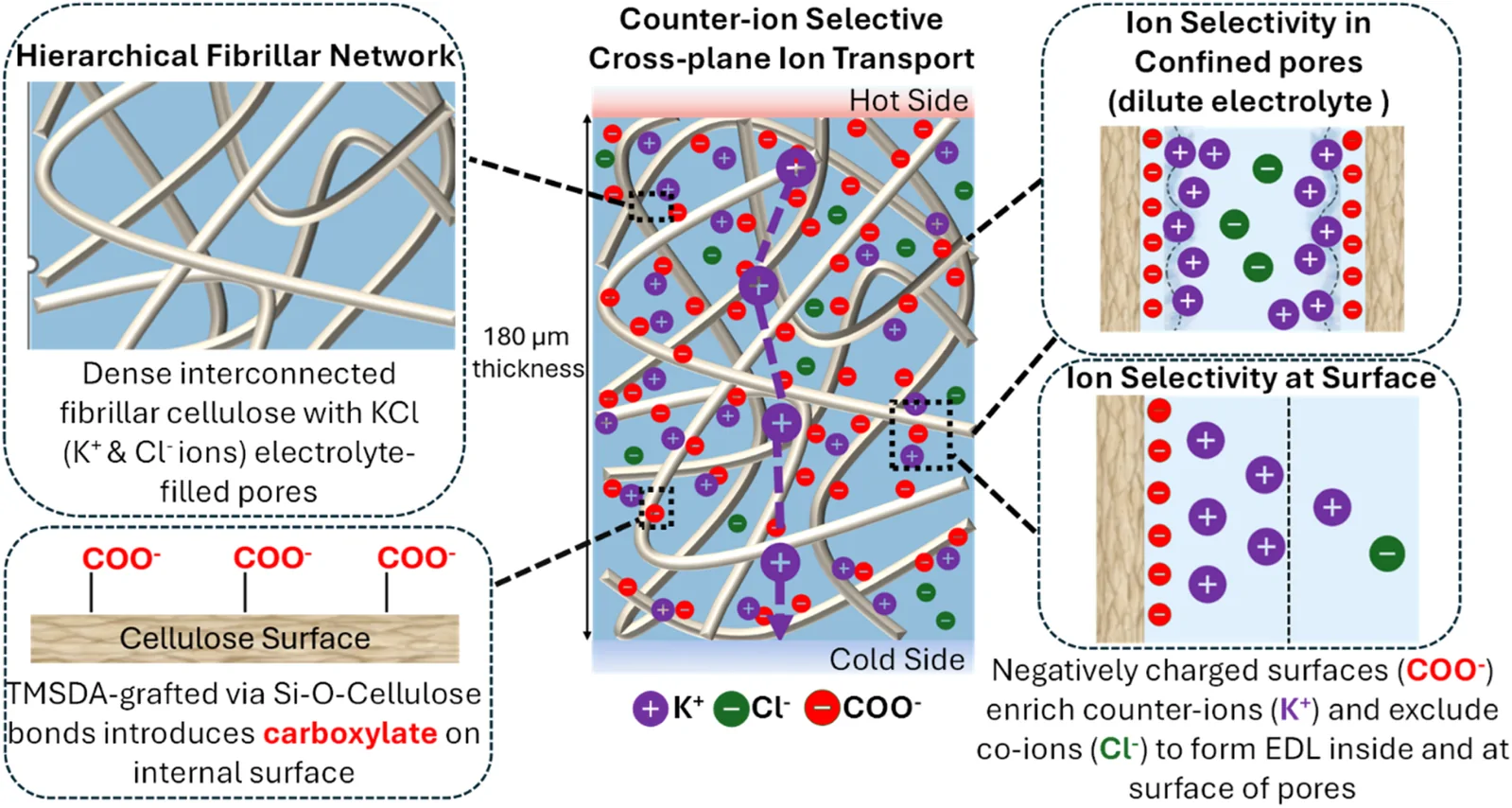
Proposed cross-plane ion transport mechanism in TMSDA-functionalised cellulose membranes. Electrolyte infiltrates the interconnected pore network formed between fibrillated cellulose domains, while TMSDA grafting introduces negatively charged carboxylate (COO^−^) groups on the internal pore surfaces. Under dilute electrolyte conditions, electric double-layer interactions enrich counter-ions (K^+^) and partially exclude co-ions (Cl^−^), promoting surface-governed ion transport through the membrane thickness. When a temperature gradient is applied across the membrane, preferential counter-ion migration generates an open-circuit thermovoltage.

The structural and surface characterisation are consistent with a hierarchical porous transport architecture containing nanoscale inter-fibrillar environments in which electrolyte infiltrates the interconnected pore network formed between fibrillated cellulose domains. At the same time, the TMSDA-derived carboxylate groups line the internal pore surfaces. Under dilute electrolyte conditions, these negatively charged interfaces enrich counter-ions and partially exclude co-ions through electric double-layer interactions. The resulting interconnected electrolyte-filled pathways provide continuous cross-plane transport channels across the membrane thickness, establishing the surface-governed ion transport mechanism illustrated in Fig. 3 and investigated experimentally in the following section.

### Ionic transport behaviour of electrolyte-infiltrated membranes

**Fig. 4:**
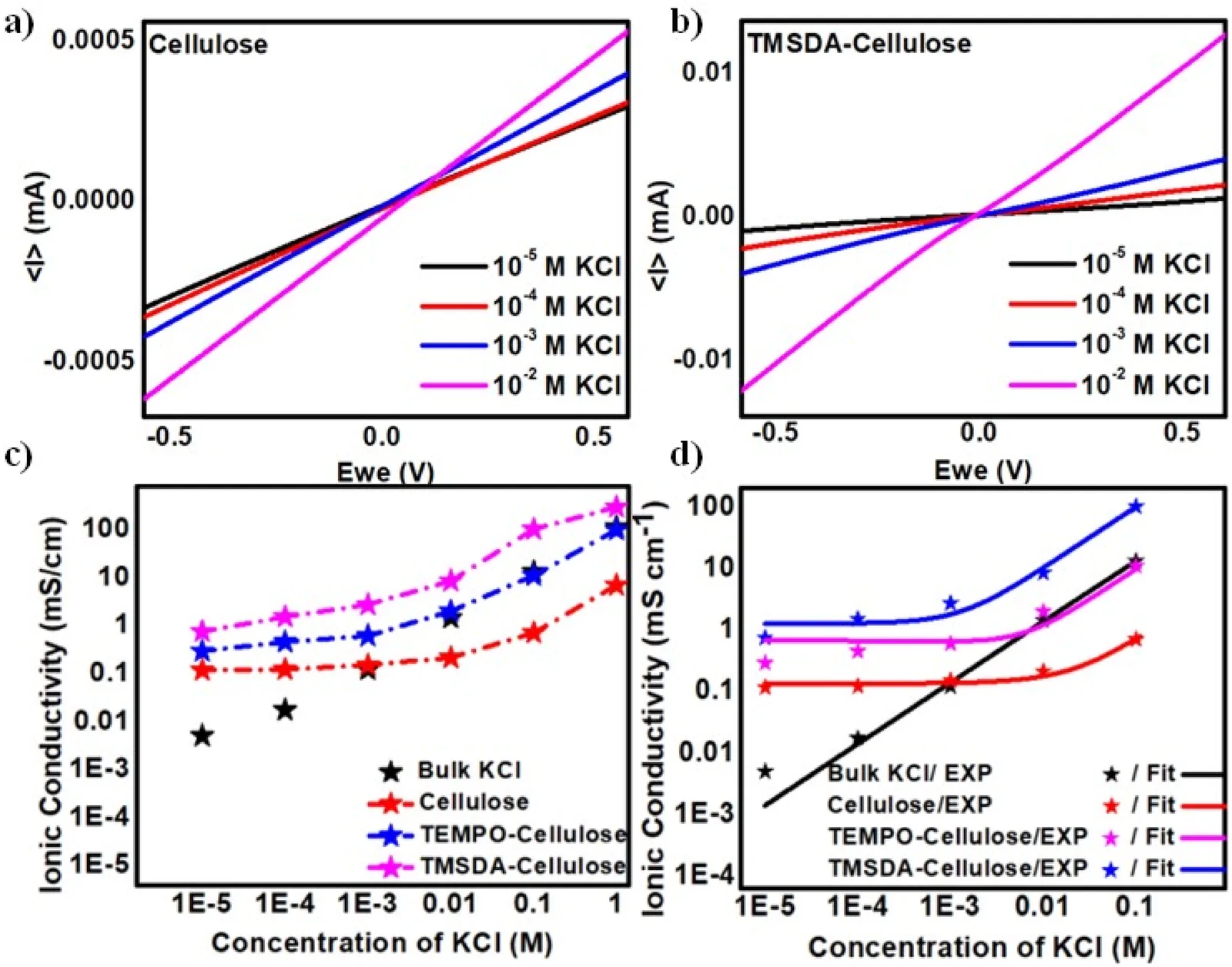
Representative LSV curves for (a) pristine cellulose and (b) TMSDA-cellulose membranes at KCl concentrations of 10^−5^ to 10^−2^ M. (c) Ionic conductivity of TEMPO-oxidised cellulose and TMSDA-cellulose compared with pristine cellulose and bulk KCl across 10^−5^ to 1 M, measured using a membrane conductivity device. (d) Experimental conductivity data for pristine cellulose, TEMPO-cellulose, TMSDA-cellulose, and bulk electrolyte systems were fitted with the model to extract conductivity parameters (eqn (3), (4), and (6)). Conductivities for membrane devices are reported as effective values calculated using eqn (2) and the macroscopic device geometry; at high ionic strength (0.1–1 M), the TMSDA-functionalised membranes can approach or exceed the bulk reference (Table S1), and *κ* should be interpreted accordingly as a membrane-in-device effective conductivity. These points were not used for extracting intrinsic surface–transport parameters.

Fixed interfacial charges on cellulose membranes play a central role in governing ion transport under confinement.^38,39^ To assess the influence of the increased fixed ionisable group content introduced by TMSDA, we measured the ionic transport properties of bulk KCl solution, pristine cellulose membranes, and TMSDA-cellulose membranes (Fig. 4). Conductivity was evaluated over a wide concentration range (10^−5^–1 M KCl). KCl was selected because it is a neutral, symmetric 1 : 1 electrolyte with well-established transport properties.^10,17,28^ Under the experimental conditions, the pH remains above the p*K*_a_ of the tricarboxylic acid groups introduced by TMSDA, ensuring that carboxylates are fully deprotonated and that surface charges are active. Ionic conductivity was determined by linear sweep voltammetry (LSV) using Ag/AgCl electrodes; representative current–voltage curves (Fig. 4(a) and (b)) are ohmic and increase monotonically with concentration.^17,27^ Across the whole range, TMSDA-cellulose carries larger currents than pristine cellulose, consistent with enhanced interfacial conduction from the grafted carboxylates.^16,38^

Fig. 4(c) compares the ionic conductivity (*κ*) of bulk KCl, pristine cellulose, TEMPO-oxidised cellulose and TMSDA-functionalised cellulose over the 10^−5^–1 M KCl concentration range. Bulk KCl exhibits the expected monotonic increase in *κ* with concentration, while pristine cellulose shows lower conductivity than the bulk solution, consistent with tortuosity and confinement within the fibre network. In contrast, TMSDA-cellulose displays an elevated low-concentration conductivity regime at ≲10^−3^ M, where *κ* exceeds the bulk KCl reference. This behaviour is consistent with surface-charge-governed transport, arising from counter-ion enrichment along the charged cellulose interfaces.^38,40^ At higher KCl concentrations, the contribution from bulk ion migration through the electrolyte-filled pore network increases and *κ* rises strongly with concentration. However, the 0.1–1 M regime should be interpreted with caution. In this high-ionic-strength range, the Debye length is very small, surface-charge effects are strongly screened, and the measured resistance becomes increasingly sensitive to the effective hydrated transport geometry, membrane/electrode contact, wetting state and possible device-level conduction pathways. Consequently, the *κ* values reported at 0.1 and 1 M are treated as apparent membrane-in-device conductivities calculated using the macroscopic device dimensions in eqn (2), rather than intrinsic membrane conductivities. The observation that TMSDA-cellulose can approach or exceed the bulk KCl reference in this regime should therefore not be interpreted as enhanced intrinsic electrolyte mobility, but rather as evidence that the simple macroscopic geometrical normalisation becomes less physically descriptive at high ionic strength. For comparison, TEMPO-oxidised cellulose exhibits conductivities that lie between pristine and TMSDA-functionalised membranes across the full concentration range (Table S1, SI). At low concentrations, TEMPO-cellulose also displays an elevated low-concentration conductivity regime relative to bulk KCl, consistent with surface-governed transport, but remains lower than TMSDA-cellulose. These trends are consistent with TEMPO-cellulose having an intermediate fixed ionisable group content relative to pristine and TMSDA-functionalised cellulose (pristine ≈ 0.16 mmol g^−1^ < TEMPO ≈ 0.31 mmol g^−1^ < TMSDA ≈ 0.86 mmol g^−1^). The systematic increase in fixed ionisable group content correlates with enhanced counter-ion enrichment and higher apparent surface conductivity, explaining the progressive elevation of the low-concentration conductivity regime.

Although both TEMPO oxidation and TMSDA functionalisation introduce negatively charged carboxylate groups onto the cellulose surface, the two strategies differ fundamentally in the number of ionisable groups introduced per modification event. TEMPO oxidation converts cellulose hydroxyl groups to carboxylate functionalities on a one-to-one basis, whereas TMSDA carries three ionisable carboxylate groups per grafted molecule. This structural difference is reflected in the titration-derived carboxylate contents: 0.86 mmol g^−1^ for TMSDA-cellulose *versus* 0.31 mmol g^−1^ for TEMPO-cellulose, corresponding to a 2.8-fold difference. The higher fixed ionisable group content of TMSDA-cellulose is expected to promote stronger electrostatic counter-ion enrichment within the confined pore network, greater electric-double-layer influence at dilute concentrations, and a nearly two-fold increase in apparent surface conductivity (*κ*_surf_ ≈ 1.26 mS cm^−1^*versus* 0.67 mS cm^−1^ for TEMPO-cellulose). This progressive enhancement, from fixed ionisable group content to interfacial transport, underpins the superior ionic thermoelectric response observed for TMSDA-cellulose relative to TEMPO-oxidised cellulose.

The interconnected fibrous network provides a hierarchical system of micro- and nanoscale transport pathways in which the interstitial regions between fibrils and fibril bundles act as confined ionic channels. At the dilute electrolyte concentrations employed in this study (≤10^−3^ M KCl), the Debye screening length is of the order of tens of nanometres, comparable to or exceeding the dimensions of the interfibrillar voids within the network. Under these conditions, electric double layers associated with the surface carboxylate groups extend across a significant fraction of the channel cross-section, promoting counter-ion enrichment and co-ion exclusion throughout the pore volume. This geometrical confinement is therefore expected to strongly enhance the contribution of surface-governed transport relative to bulk electrolyte conduction, a prediction supported by the elevated low-concentration conductivity regime, the extracted surface conductivity *κ*_surf_, and the millimolar crossover concentration c* obtained from the transport analysis in subsequent sections.

To quantitatively distinguish interfacial (surface) contributions from intrinsic electrolyte behaviour within the membranes, it is first necessary to establish a reliable bulk reference. We therefore modelled the conductivity of KCl in the absence of confinement using the classical Kohlrausch relation for strong electrolytes (eqn (3) and (4)).^41–43^ In this framework, the molar conductivity *Λ*_m_(*c*) is expressed as3
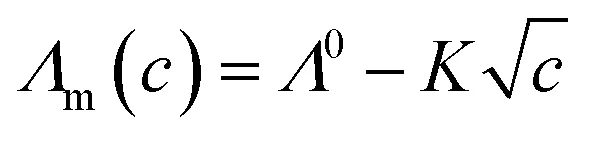


and the corresponding bulk conductivity is

4 *κ*_bulk_(*c*) = *Λ*_m_(*c*)*c*

where *Λ*_m_(*c*) is the molar conductivity at concentration *c*, *Λ*^0^ is the limiting molar conductivity (S cm^2^ mol^−1^), K is an empirical coefficient (S cm^2^ mol^−1^ M^−1/2^) that accounts for ion–ion interactions at finite concentration, and *κ*_bulk_(*c*) is the bulk contribution to the conductivity. Fitting the bulk KCl conductivity data yielded *Λ*^0^ ≈ 141 S cm^2^ mol^−1^ and *K* ≈ 28.2 S cm^2^ mol^−1^ M^−1/2^, values that are in good agreement with literature reports for KCl at near-room temperature.^44^ Minor deviations can be attributed to small temperature offsets, uncertainties in the cell constant/geometry and the broad concentration window used for fitting. Overall, the Kohlrausch relation accurately describes our bulk KCl dataset and provides a reliable reference for separating bulk and interfacial contributions in the membrane systems.

For ion transport in charged, electrolyte-filled pores, the total conductivity can be written as the sum of a bulk contribution, which scales with the electrolyte concentration, and a surface contribution arising from counter-ions associated with the charged pore walls. For a symmetric 1 : 1 electrolyte, this behaviour is commonly described using the expression shown in eqn (5):^17,45^5
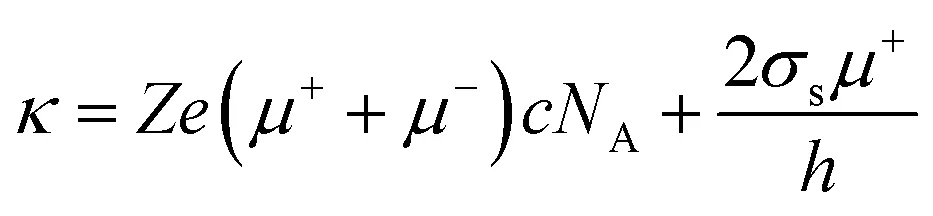
where *Z* is the cation valence, *e* is the elementary charge, *µ*^+^ and *µ*^−^ are the cation and anion mobilities, *c* is the bulk electrolyte concentration, *N*_A_ is Avogadro's number, *σ*_s_ is the physical area-normalised surface charge density at the pore wall, and *h* is an effective channel dimension. Here, *σ*_s_ should be distinguished from the titration-derived fixed ionisable group content reported above in mmol g^−1^. The latter provides an experimental measure of carboxylate loading within the cellulose membrane, whereas *σ*_s_ represents an area-normalised surface charge density in the transport expression. Because the internal pore surface area and effective channel dimensions are not independently measured in this work, eqn (5) is used as a conceptual framework for understanding the origin of surface-governed conductivity, rather than to calculate *σ*_s_ from the titration values directly. Quantitative comparison between membranes is therefore made using the experimentally fitted apparent surface conductivity, *κ*_surf_, and crossover concentration, c*.

To capture this surface-to-bulk crossover quantitatively and to compare different membranes on a similar footing, we modelled the total conductivity *κ*(*c*) as the sum of a bulk term and an interfacial term, see eqn (6):^38,46,47^6
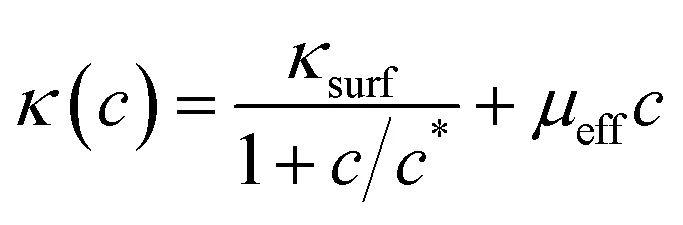
where *κ*(*c*) is the measured total conductivity (mS cm^−1^), *κ*_surf_ is the low-concentration surface conductivity (mS cm^−1^), c* is the crossover concentration that marks the transition from surface-to-bulk-dominated transport, and *µ*_eff_ is an effective mobility parameter that captures the bulk-like contribution within the pores. In eqn (6), the first term describes the concentration-dependent surface contribution, while the *µ*_eff_*c* term captures the bulk-like contribution within the electrolyte-filled pore network.Eqn (3) and (4) describe the bulk KCl reference, whereas eqn (6) is used as a phenomenological fit to the membrane conductivity data. Thus, *κ*_surf_ provides an experimentally fitted measure of the apparent surface contribution to ion transport, rather than a directly calculated intrinsic surface conductivity.

Table S2 summarises the parameters extracted by fitting eqn (6) to the pristine and TMSDA-cellulose conductivity data. A TEMPO-oxidised cellulose comparator yields an intermediate surface conductivity (*κ*_surf_ ≈ 0.67 mS cm^−1^) and crossover concentration (c* ≈ 5 × 10^−3^ M), lying between pristine and TMSDA-functionalised cellulose (see Table S2). Pristine cellulose exhibited a small surface conductivity, *κ*_surf_ ≈ 0.13 mS cm^−1^, consistent with its modest density of ionisable surface groups and weak negative interfacial charge. Upon introducing the tricarboxylate TMSDA moiety, *κ*_surf_ increased by approximately an order of magnitude to ≈ 1.26 mS cm^−1^, in line with the 5.4-fold increase in titration-derived carboxylate content and the more negative *ζ*-potential. The fitted crossover concentration c* lies in the low-millimolar range, indicating that TMSDA-cellulose remains in a surface-dominated transport regime at the lowest concentrations studied. For all fits, we restricted the concentration range to 10^−5^–10^−2^ M, which captures the elevated low-concentration conductivity regime and the onset of the surface-to-bulk crossover that the model is intended to describe. At higher ionic strengths (≥0.1 M), *κ* should be treated as an effective membrane-in-device conductivity under the macroscopic geometrical normalisation (eqn (2)), and can be influenced by changes in the effective hydrated transport pathway; accordingly, including the 1 M points biases the optimisation and can force parameter values that are not representative of the low-to-intermediate concentration regime. Overall, the fitted trends support that TMSDA grafting substantially enhances interfacial conduction and that, under dilute conditions, ion transport in these membranes is strongly influenced by interfacial charge.

### EIS and validation of LSV-derived conductivities

To independently validate the ionic conductivities extracted from LSV, EIS was performed on the cellulose paper membranes over the concentration range 10^−5^–10^−2^ M KCl. Impedance spectra were recorded under the same cell geometry and using the same membranes as in the LSV measurements, ensuring that both techniques probed the same effective transport path.

Representative Nyquist plots display a single, well-defined single semicircle over 10^−5^–10^−2^ M KCl, with no evidence of additional low-frequency features such as Warburg diffusion tails (see Fig. S6 in SI). The spectra were therefore fitted using a simple two-element equivalent circuit of the form, as shown in eqn (7):7
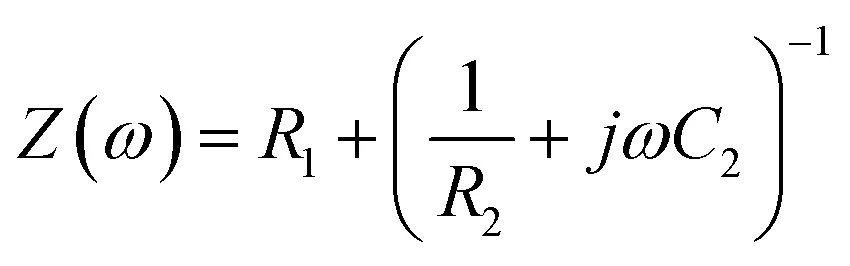
where *R*_1_ represents the series resistance of the electrolyte and cell leads, *R*_2_ is the membrane (or interfacial) resistance, and *C*_2_ is an effective interfacial/membrane capacitance. This is the minimal model capable of reproducing the observed single semicircle, and attempts to introduce more complex circuits (constant-phase elements, additional RC branches, or diffusion elements) did not appreciably improve the quality of the fits.

The fitted parameters for the cellulose samples are summarised in Table S3. Across three orders of magnitude in KCl concentration, *C*_2_ remains nearly constant at ≈ 18–22 pF; consistent with a largely geometric/effective capacitance associated with the wetted pore network and double layer, while *R*_2_ decreases monotonically from 3.1 × 10^5^ Ω at 10^−5^ M to 4.1 × 10^4^ Ω at 10^−2^ M. The modest variation in *R*_1_ (672–960 Ω) is attributed to small run-to-run changes in wiring and contact resistance and has a negligible impact on the extracted membrane resistance.

For each concentration, an “AC conductivity” was calculated from the fitted *R*_2_ values *via*eqn (8):8
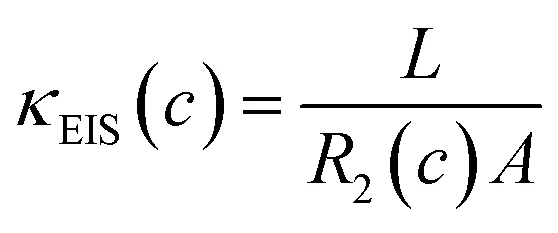
where *L* is the membrane thickness, and *A* is the active membrane area. These *κ*_EIS_ values were compared directly with the “DC” conductivities *κ*_LSV_ obtained from the slope of the low-bias region of the LSV I–V curves, using the same membrane thickness and active area. Over the range 10^−5^–10^−2^ M KCl, *κ*_EIS_ and *κ*_LSV_ agree within the experimental uncertainty of the measurements (of order 20–30%), and both methods yield the same monotonic increase in conductivity with salt concentration. Importantly, there is no systematic offset between the AC and DC conductivities: at each concentration, the LSV-derived values lie on the trend defined by the EIS-derived values.

This agreement indicates that (i) the LSV experiments were performed within a linear, ohmic regime (no obvious evidence of electrode polarisation or mass-transport limitation in the potential window used), and (ii) the simple geometrical correction used to convert LSV currents into conductivities is appropriate. The EIS measurements, therefore, serve as an independent cross-check, supporting the reliability of the LSV-based conductivity data that underpin the subsequent surface-bulk transport analysis.

### Behaviour at high ionic strength (0.1 M and 1 M KCl)

At 0.1 M and 1 M KCl, the Nyquist response differs qualitatively from the lower-concentration spectra. The overall impedance is small, and the spectra consist of a shallow, strongly compressed arc with only a partial semicircle visible within the instrument's accessible frequency window. In this regime, the membrane resistance becomes comparable to, and strongly masked by, the series resistance of the highly conducting electrolyte. Because the lower frequency limit of the EIS measurements does not extend far enough to capture the complete low-frequency intercept of the semicircle, the fitted *R*_2_ and *C*_2_ values at 1 M are poorly constrained and sensitive to the chosen equivalent-circuit model.

We therefore do not use the 1 M EIS data for quantitative analysis or conductivity extraction. The conductivity value reported at 1 M is instead derived from the low-bias region of the LSV I–V response and is presented only as an apparent membrane-in-device conductivity. Reliable impedance spectra could not be obtained at 0.1 M KCl under our measurement conditions, so the conductivity at this concentration is also reported from LSV alone. As the 0.1–1 M regime corresponds to high ionic strength, where surface-charge effects are strongly screened, and the measured resistance becomes increasingly sensitive to wetting, contact resistance, and effective device geometry, these values were not used to extract intrinsic transport parameters or to support the surface-governed transport model. In this regime, TMSDA-functionalised membranes can exhibit *κ* values that approach or exceed the bulk KCl reference; such values should therefore be interpreted as apparent conductivities calculated using the macroscopic geometrical normalisation in eqn (2), rather than as intrinsic electrolyte or membrane conductivities.

Taken together, the robust EIS-LSV agreement over 10^−5^–10^−2^ M supports the reliability of the LSV-derived conductivities in the low-to-intermediate concentration range used for surface-bulk transport analysis. At higher ionic strengths, the LSV-derived values are treated as apparent membrane-in-device conductivities.

Full details of the equivalent circuit, fitting procedure, and extracted parameters (*R*_1_, *R*_2_, *C*_2_) are given in Table S3 and as a subsection in SI.

### Counter-ion identity (Na^+^ carryover) and robustness

During TMSDA silanisation, the tricarboxylate silane is introduced as its sodium salt, so sodium counter-ions (Na^+^) are initially present to balance the deprotonated carboxylate groups. Even after extensive rinsing with deionised water, a fraction of these Na^+^ ions may remain associated with the grafted carboxylates or within the pore network before the subsequent KCl conductivity measurements. Such residual Na^+^ could slightly alter the apparent surface conductivity, *κ*_surf_, because Na^+^ and K^+^ have different limiting molar ionic mobilities.

To evaluate this potential artefact, a mobility-weighted mixing correction was applied using the limiting molar ionic mobilities at 25 °C (*µ*_K^+^_ ≈ 73.5, *µ*_Na^+^_ ≈ 50.1 S cm^2^ mol^−1^).^48^ The scaling factor, *S*_mix_, accounts for the fractional presence of Na^+^ (*f*_Na_) and is defined in eqn (9):^46,49,50^9
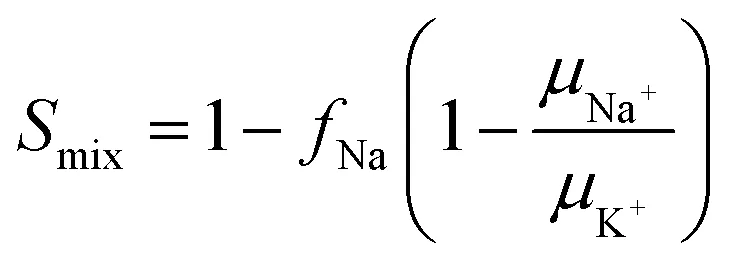


The corrected surface conductivity was then expressed as eqn (10):10
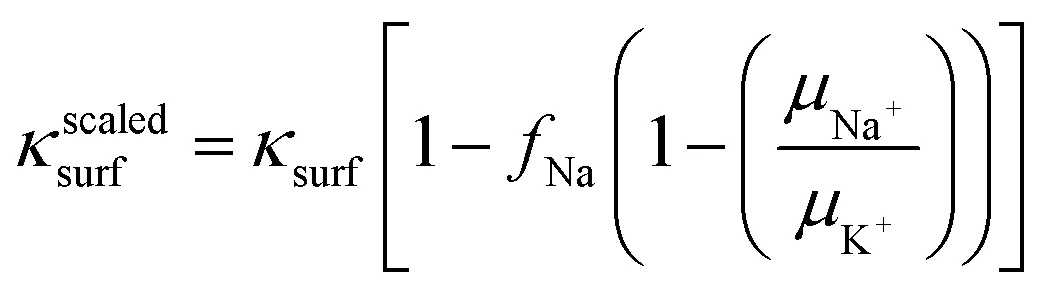


As shown in SI Fig. S7, the analysis was performed for *f*_Na_ = 0–30%, representing plausible levels of residual Na^+^ after rinsing. Across this range, the scaled *κ*_surf_ decreased by less than 10%, while both the effective concentration-scaling coefficient (*µ*_eff_) and the crossover concentration (c*) remained essentially unchanged. The low-concentration conductivity plateau also remained in close agreement with the experimental data, indicating that the surface-bulk conductivity model is not sensitive to moderate Na^+^ contamination.

To further validate the robustness of the surface-conduction model, identical analyses were performed on pristine cellulose membranes that had never been exposed to sodium reagents. The resulting overlays (Fig. S7(a)) exhibited negligible deviations, indicating that the small ionic-mobility mismatch between Na^+^ and K^+^ has a minor quantitative impact and no effect on the qualitative trends or physical interpretation of *κ*_surf_, c*and *µ*_eff_.^38,45^ Hence, the extracted fitting parameters and the inferred enhancement in surface-governed ionic conduction for TMSDA-cellulose are not strongly affected within the assumed range of residual Na^+^ fractions. For contextual benchmarking, Table S4 (see SI) compares the extracted fitting parameters, including *κ*_surf_, *µ*_eff_, and c*, for pristine and TMSDA-functionalised cellulose membranes with representative cellulose-based nanofluidic systems reported in the literature. The notably higher *κ*_surf_ achieved here underscores the dominant role of surface-charge enhancement *via* TMSDA grafting in facilitating efficient interfacial ion transport.

### Ionic thermoelectric properties of the membranes

Building on the surface-governed transport behaviour established above, we next examined how the increased fixed ionisable group content and stronger interfacial charge of TMSDA-cellulose translate into ionic thermoelectric performance. The conductivity analysis (Fig. 4(c) and (d)) shows that at 10^−4^ M KCl the membranes operate below the crossover concentration c*, in a regime where ion transport is expected to have a strong surface-governed/counter-ion contribution. To exploit this, all thermoelectric measurements were performed in 10^−4^ M KCl, where strong ion selectivity and interfacial transport are expected to maximise the ionic Seebeck coefficient.

The ionic thermoelectric performance of the membranes was assessed in a sandwich-cell configuration, in which cellulose membranes were infiltrated with 10^−4^ M KCl and clamped between two gold-coated silicon wafer electrodes. To obtain sufficiently large thermovoltages while retaining a short thermal path length, we assembled stacks of five TMSDA-cellulose membranes; pristine cellulose stacks with the same geometry were used as references. Before measurement, the membrane stacks were thoroughly wetted by soaking in 10^−4^ M KCl, a concentration that lies within the surface-governed regime (*c* < c*), ensuring that the negatively charged cellulose surfaces strongly influence ion transport. After equilibration, excess solution was removed, and the saturated stacks were transferred to the thermoelectric cell for characterisation.^30^

**Fig. 5:**
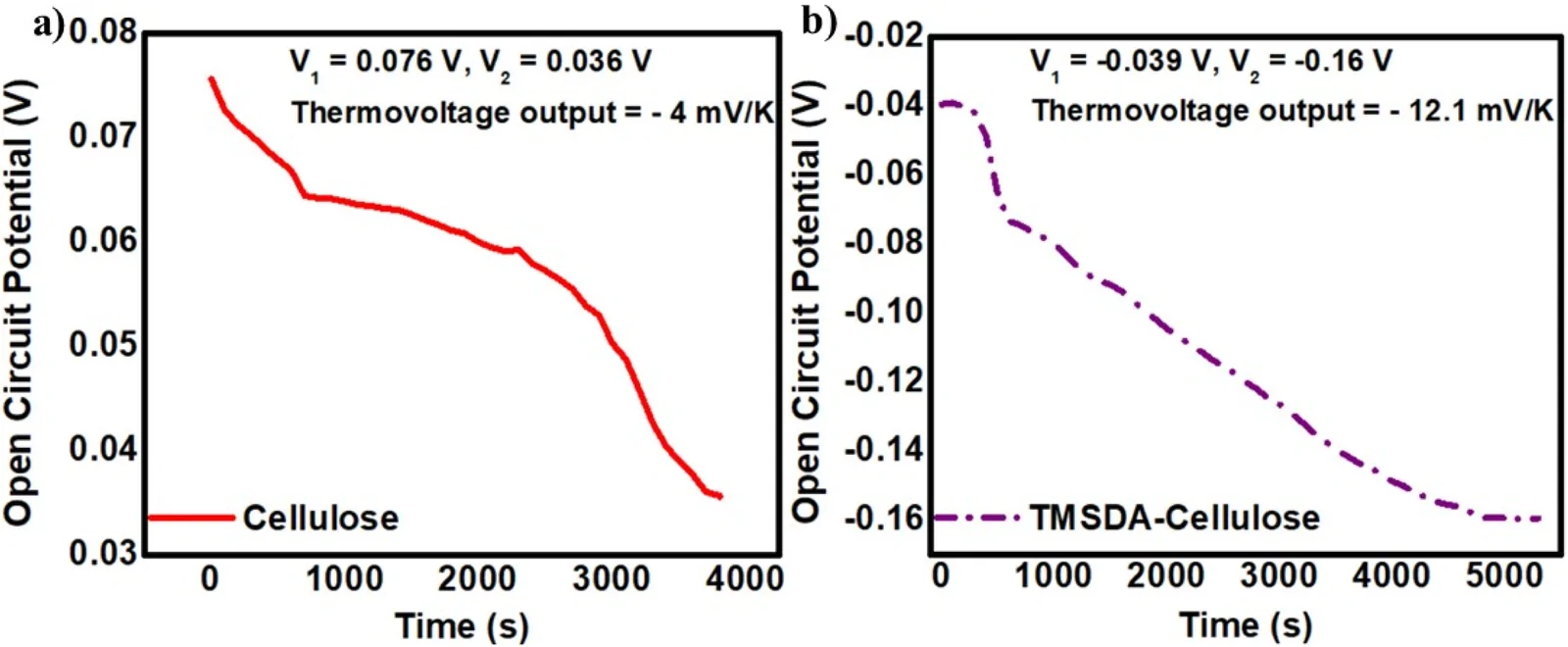
Thermal charging behaviour of 10^−4^ M KCl-infiltrated cellulose membranes measured in a sandwich configuration between two gold-coated silicon wafer electrodes: (a) pristine cellulose and (b) TMSDA-functionalised cellulose. The enhanced apparent single-step ionic thermovoltage for TMSDA-cellulose corresponds to an apparent ionic Seebeck coefficient that is ∼3.2-fold higher than that of pristine cellulose (10^−4^ M KCl, Δ*T* = 10 K).

A constant temperature gradient (Δ*T*) of 10 K was applied across the membrane stack by maintaining one electrode at a higher temperature than the other (see Experimental Section and Fig. S8 in SI). The open-circuit voltage (*V*_oc_) and the apparent ionic Seebeck coefficient were obtained as *S*_i_ = *V*_oc_/Δ*T* for this 10 K step. Stacked pristine cellulose membranes infiltrated with 10^−4^ M KCl exhibited an apparent ionic Seebeck coefficient of about −4 mV K^−1^, whereas TMSDA-cellulose stacks yielded a substantially larger coefficient of −12.8 ± 1.76 mV K^−1^ (Fig. 5). This ∼3.2-fold increase in *S*_i_, together with the enhanced conductivity of TMSDA-cellulose, resulted in an ionic thermoelectric power factor of 24.7 ± 6.8 µW m^−1^ K^−2^ at Δ*T* = 10 K for the TMSDA-cellulose stack, ranking among the highest values reported for neutral aqueous cellulose-based systems. Because the measurements were performed at a single temperature step, these ionic Seebeck coefficients are best regarded as apparent, single-point values, assuming a linear response over 0–10 K. TEMPO-oxidised cellulose stacks showed an intermediate apparent thermopower of approximately −5 mV K^−1^ (see SI Fig. S8).

The lower apparent thermopower of TEMPO-cellulose (approximately −5 mV K^−1^) relative to TMSDA-cellulose (−12.8 mV K^−1^) is directly consistent with its lower fixed ionisable group content (0.31 *vs.* 0.86 mmol g^−1^) and reduced apparent surface conductivity (*κ*_surf_ ≈ 0.67 *vs.* 1.26 mS cm^−1^). The lower fixed ionisable group content of TEMPO-cellulose leads to less effective counter-ion enrichment and co-ion exclusion within the confined pore network, reducing the mobility asymmetry between K^+^ and Cl^−^ under the applied temperature gradient and thereby producing a smaller thermovoltage. The systematic progression in thermoelectric response across pristine (approximately −4 mV K^−1^), TEMPO-oxidised (approximately −5 mV K^−1^), and TMSDA-functionalised (−12.8 mV K^−1^) cellulose membranes directly mirrors the progressive increase in fixed ionisable group content and apparent surface conductivity established in the transport analysis, supporting fixed ionisable group content and surface-governed transport as major contributors to ion selectivity, thermodiffusion asymmetry, and ionic thermoelectric performance in these systems.

The ionic Seebeck coefficient in nanofluidic membranes is governed by the Soret effect, in which cations and anions thermodiffuse at different rates in response to a temperature gradient.^51^ Charged porous/inter-fibrillar pathways preferentially accumulate counterions while excluding co-ions, creating a mobility asymmetry that enhances thermopower.^10^ This principle has been widely demonstrated. For instance, aligned cellulose nanofibre membranes with titration-derived carboxylate contents of approximately 0.16 mmol g^−1^ before oxidation and 0.25 mmol g^−1^ after TEMPO oxidation displayed enhanced Na^+^ selectivity and repulsion of OH^−^ anions, producing a thermopower of ∼24 mV K^−1^ in 0.65 M NaOH-polyethylene oxide.^16^ Similarly, when functionalised with a positively charged quaternary ammonium group, CHMAC-functionalised regenerated cellulose membranes achieved an ionic Seebeck coefficient of +6.1 mV K^−1^ in dilute HCl, a tenfold improvement over unmodified cellulose, under the same conditions.^27^ Together, these studies support a strong relationship between fixed ionisable group content, ion selectivity and ionic thermopower. Similarly, in our system, the ∼5.4-fold increase in titration-derived carboxylate content translates into a strong negative electrostatic field that attracts K^+^ and repels Cl^−^ ions, amplifying the difference in thermodiffusion mobilities and yielding a ∼3.2-fold rise in the effective ionic Seebeck coefficient (−4 to −12.8 mV K^−1^, normalised from the thermovoltage measured under a 10 K gradient). This mechanistic framework explains the high thermovoltage response of the TMSDA-cellulose stacks, emphasising the role of surface-charge-mediated thermodiffusion in ionic thermoelectrics.

Temperature-dependent ionic conductivity measurements further support this interpretation (see Fig. S9 in SI). The Arrhenius dependence of conductivity, shown in eqn (11):11
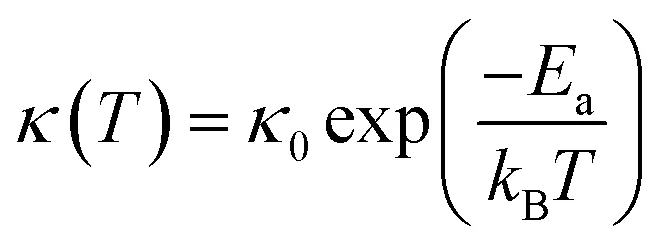
where *κ*_0_ is the pre-exponential factor, *E*_a_ is the apparent activation energy, and *k*_B_ is the Boltzmann constant.^16,29^ This model enables the extraction of *E*_a_ for ion migration, reflecting the energy barrier associated with ion transport. For TMSDA-cellulose, an *E*_a_ of 0.37 eV was obtained (Fig. S9(b)). This intermediate value is higher than that of bulk aqueous diffusion but lower than that of dense solid electrolytes,^52–57^ suggesting that the functionalised cellulose matrix facilitates ionic transport while remaining influenced by nanoscale confinement and surface interactions. The thermally activated increase in conductivity is consistent with thermally activated ionic transport contributing to the observed thermovoltage response. However, the apparent ionic Seebeck coefficient also depends on thermodiffusive asymmetry and ion selectivity.

### Comparison with reported ionic thermoelectric membranes

To place the performance of the TMSDA-cellulose membranes in context, Table 1 summarises representative ionic thermoelectric membrane systems reported in the literature, alongside those in the present work. The table compares key figures of merit, including the ionic Seebeck coefficient and conductivity, as well as the membrane composition, electrolyte medium, and operating conditions. Many of the highest reported power factors are achieved in systems that employ dense polyelectrolytes, ionic liquids or strongly acidic/alkaline electrolytes, often combined with complex processing routes or non-renewable polymer backbones.^16,58–62^

**Table 1:** Comparative performance of representative ionic thermoelectric membrane and hydrogel systems reported in the literature

Membrane system	Electrolyte/environment	Thermo-output (mV K^−1^)	Ionic conductivity (mS cm^−1^)	Sustainability notes
Wood-chip cellulose, TMSDA-functionalised (this work)	Dilute aqueous KCl (neutral)	−12.8	1.51	Waste-derived, biodegradable; simple, green chemistry
Bacterial cellulose-BN composite^58^	Artificial seawater (0.5 M NaCl) *vs.* river water (0.01 M NaCl)	0.52–0.74	∼1	Biodegradable cellulose;^63^ BN is synthetic and non-biodegradable^64^
NFC-PSSNa composite paper^59^	Humid environment (100% RH)	8.4	9	Renewable NFC; PSSNa petroleum-derived, not biodegradable^65^
Aligned cellulose nanofibre, TEMPO-oxidised^16^	PEO-water-0.65 M NaOH	24	20	Wood-derived; relies on concentrated alkaline electrolyte
MOF – polymer ionogel (IL + ZIF-8)^60^	Ionic liquid (EMIM:DCA) + 40 wt% ZIF-8, 80% RH	31.9	18.8	High performance; MOF + IL non-renewable, non-biodegradable.^66–69^
Ca^2+^-crosslinked bacterial cellulose hydrogel^61^	Water-rich hydrogel (Na^+^/Cl^−^)	−27.2	204.2	Sustainable feedstock: however, bacterial cellulose production is costly and energy intensive.^70^ hydrogels face stability and scalability issues^71^
PVA + CsI hydrogel^62^	Hydrogel (0.1 M CsI)	52.9	∼10	PVA partially biodegradable; CsI scarce and toxic.^72,73^

The TMSDA-functionalised cellulose membranes developed in this work are prepared from industrial wood waste using a simple aqueous silane functionalisation and operate in a benign neutral 10^−4^ M KCl electrolyte. Under these conditions, the stacked membranes exhibit an apparent ionic Seebeck coefficient of −12.8 ± 1.76 mV K^−1^ and a power factor of 24.7 ± 6.8 µW m^−1^ K^−2^ at Δ*T* = 10 K, placing their performance within the upper range reported for cellulose-based and other polymeric ionic thermoelectric membranes operating in aqueous media. Beyond the thermoelectric performance itself, the present study establishes a quantitative relationship between fixed ionisable group content, surface conductivity (*κ*_surf_), the crossover concentration (c*), and the resulting ionic thermoelectric response through a surface–bulk transport model. This mechanistic analysis provides direct experimental evidence that increasing the density of fixed ionisable groups enhances counter-ion enrichment and interfacial ion transport, thereby improving ionic thermoelectric performance.

Compared with previously reported cellulose-based ionic thermoelectric materials, the present approach offers several practical advantages. High-performing systems based on TEMPO-oxidised aligned cellulose nanofibres (*S*_i_ ∼24 mV K^−1^) and Ca^2+^-crosslinked bacterial cellulose hydrogels (*S*_i_ ∼ −27.2 mV K^−1^) achieve superior thermopower, but require concentrated alkaline electrolytes or ionic liquid salt additives, costly bacterial cellulose feedstocks, or hydrogel architectures that face mechanical robustness and scalability limitations.^16,61^ Similarly, TEMPO-oxidised carboxylated bacterial cellulose hydrogels doped with LiTFSI deliver *S*_i_ ∼11.53 mV K^−1^ but again rely on non-neutral electrolyte conditions and hydrogel formats.^23^ TMSDA functionalisation introduces a substantially higher fixed ionisable group content (0.86 mmol g^−1^) than TEMPO oxidation (0.31 mmol g^−1^) while preserving a simple porous cellulose membrane architecture derived from renewable waste biomass and operating entirely in a neutral aqueous electrolyte. The combination of sustainable feedstock, environmentally benign processing and mechanistic understanding therefore distinguishes the present charge-engineering strategy from previously reported cellulose-based ionic thermoelectric membranes.

Beyond demonstrating competitive thermoelectric performance, the developed TMSDA-cellulose membranes also exhibit characteristics that are attractive for practical ionic thermoelectric devices. The combination of a renewable, waste-derived cellulose scaffold, simple aqueous processing, and operation in a neutral KCl electrolyte provides clear advantages over systems based on concentrated alkaline media, ionic liquids or synthetic polymer matrices, which may present challenges in terms of environmental compatibility, long-term stability and scalability. At a modest temperature difference of only 10 K, the stacked membranes generate an open-circuit thermovoltage of approximately −128 mV, demonstrating their ability to convert low-grade thermal gradients directly into measurable electrical signals. Such temperature differences are commonly found on industrial equipment, building envelopes, and in ambient environmental fluctuations, suggesting opportunities to harvest otherwise wasted thermal energy. Furthermore, the relationship between thermovoltage and temperature difference suggests potential for self-powered thermal sensing. At the same time, the modular stacked-membrane architecture offers a straightforward route to increase voltage output by adding membrane layers or series-connected cells. These features highlight the practical potential of charge-engineered, waste-derived cellulose membranes as sustainable platforms for ionic thermoelectric energy harvesting and sensing technologies.

**Fig. 6:**
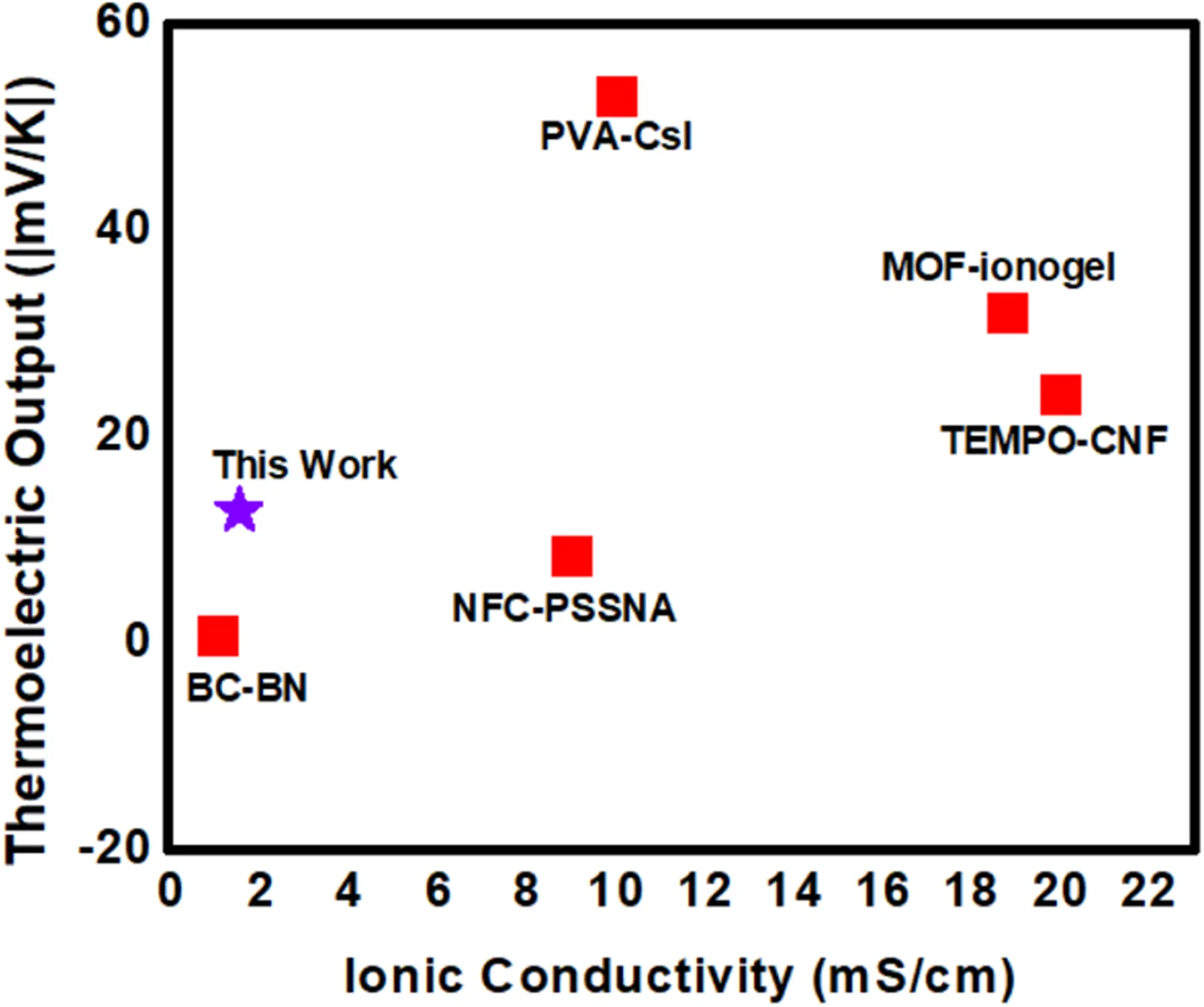
Comparison of the absolute ionic Seebeck coefficient, |*S*_i_|, as a function of ionic conductivity for representative membrane-based ionic thermoelectric systems and the TMSDA-functionalised cellulose membrane developed in this work. Absolute values are plotted to compare thermoelectric output magnitude across systems with different voltage polarities.

To further compare performance, Fig. 6 plots the absolute ionic Seebeck coefficient |*S*_i_| as a function of ionic conductivity for representative membrane-based ionic thermoelectric materials. The TMSDA-functionalised cellulose membrane occupies a favourable region of the performance space, combining relatively high thermopower with moderate ionic conductivity despite operating under dilute neutral electrolyte conditions. This behaviour reflects operation within the surface-governed transport regime (*c* < c*), where enhanced counter-ion confinement produces a large thermoelectric response without requiring highly conductive ionic liquids or concentrated electrolytes. Collectively, the literature comparison demonstrates that combining a porous nanostructured scaffold with a high content of fixed ionisable groups and strong interfacial charge is an effective design strategy for achieving high ionic thermoelectric performance while maintaining environmentally benign processing and operation.

## Conclusion

This work demonstrates that charge-engineered cellulose membranes derived from waste wood chips are effective ionic thermoelectric materials under environmentally benign conditions. Mild aqueous functionalisation with TMSDA significantly increases the negative surface charge and zeta potential while maintaining the structural integrity of the membranes, promoting counter-ion confinement and surface-governed ion transport at dilute electrolyte concentrations (*c* ≪ c*). Conductometric titration shows a ∼5.4-fold increase in fixed ionisable group content after TMSDA functionalisation, while concentration-dependent conductivity analysis reveals an approximately order-of-magnitude enhancement in apparent surface conductivity and identifies a millimolar crossover concentration marking the transition from surface- to bulk-dominated transport. When stacked and infiltrated with 10^−4^ M KCl, TMSDA-cellulose membranes generate an apparent ionic Seebeck coefficient of −12.8 ± 1.76 mV K^−1^ and an ionic thermoelectric power factor of 24.7 ± 6.8 µW m^−1^ K^−2^, corresponding to a ∼3.2-fold improvement in apparent thermopower over pristine cellulose and placing their performance among the higher reported values for cellulose-based ionic thermoelectrics operating in dilute neutral aqueous electrolytes. Beyond their fundamental significance, the TMSDA-cellulose membranes exhibit attributes directly relevant to practical ionic thermoelectric applications. The combination of a waste-derived feedstock, simple aqueous processing, mechanical robustness, and operation in a benign neutral electrolyte offers clear advantages over systems relying on ionic liquids, concentrated alkaline media, or synthetic polymer matrices, positioning these membranes as practical candidates for low-grade waste heat harvesting from industrial and environmental sources and for self-powered thermal sensing applications. The modular stacked-membrane architecture demonstrated here also provides a straightforward route to scaling voltage output for real-world energy harvesting. More broadly, the design principles established in this work, quantitative charge engineering, surface–bulk transport modelling, and operation in the surface-dominated regime, provide a transferable framework for developing next-generation sustainable nanofluidic membranes for ionic thermoelectric and related electrochemical energy conversion technologies.

While the present work focuses on cross-plane measurements in a single neutral electrolyte at a fixed temperature gradient, the design rules identified here are general. Future studies could explore in-plane device geometries, alternative counter-ions and mixed electrolytes, and other cellulose sources or silane chemistries to tune further the balance between the ionic Seebeck coefficient, conductivity, and long-term stability. Such efforts will help translate the charge-engineered, waste-derived cellulose platforms demonstrated here into practical ionic thermoelectric modules and other sustainable electrochemical devices.

## Conflicts of interest

There are no conflicts to declare.

## Supplementary Material

RA-016-D6RA03519A-s001

## Data Availability

The datasets generated and/or analysed during the current study are available in the TRANSLATE Zenodo repository at https://zenodo.org/communities/translateh2020/. Supplementary information (SI): cellulose extraction and TMSDA functionalisation; membrane morphology and stability; electrochemical and ionic thermoelectric measurement setups; ionic conductivity data, modelling and EIS analysis; literature benchmarking; thermovoltage measurements; and temperature-dependent conductivity and Arrhenius analysis. See DOI: https://doi.org/10.1039/d6ra03519a.
