# Supplementary material for: Charge-engineered cellulose membranes from wood waste for ionic thermoelectric energy conversion
Source: RSC Adv. 2026 Aug 5;16(39):44909–23. doi: 10.1039/d6ra03519a (PMC13440344; doi:10.1039/d6ra03519a)
Supplement: Supplementary file 1 [file RA-016-D6RA03519A-s001.pdf]

Supplementary Information (SI) for RSC Advances.
This journal is © The Royal Society of Chemistry 2026

Supporting Information

Charge-Engineered Cellulose Membranes from Wood Waste for Ionic Thermoelectric Energy Conversion

Anjali Ashokan[1,2,3], Rupa Ranjani Palanisamy[1,3], Kafil M. Razeeb[3], Subhajit Biswas*[1,2] and Justin D. Holmes*[1,2]

[1]*School of Chemistry, University College Cork, Cork, T12 YN60, Ireland.*

[2]*AMBER Centre, Sustainability Institute, University College Cork, Cork, T23 XE10, Ireland.*

[3]*Micro-Nano System Centre, Tyndall National Institute, Cork, T12 R5CP, Ireland.*

*Corresponding authors: s.biswas@ucc.ie or j.holmes@ucc.ie

Keywords: Cellulose membranes, waste valorisation, surface functionalisation, ionic thermoelectrics, sustainable energy harvesting.

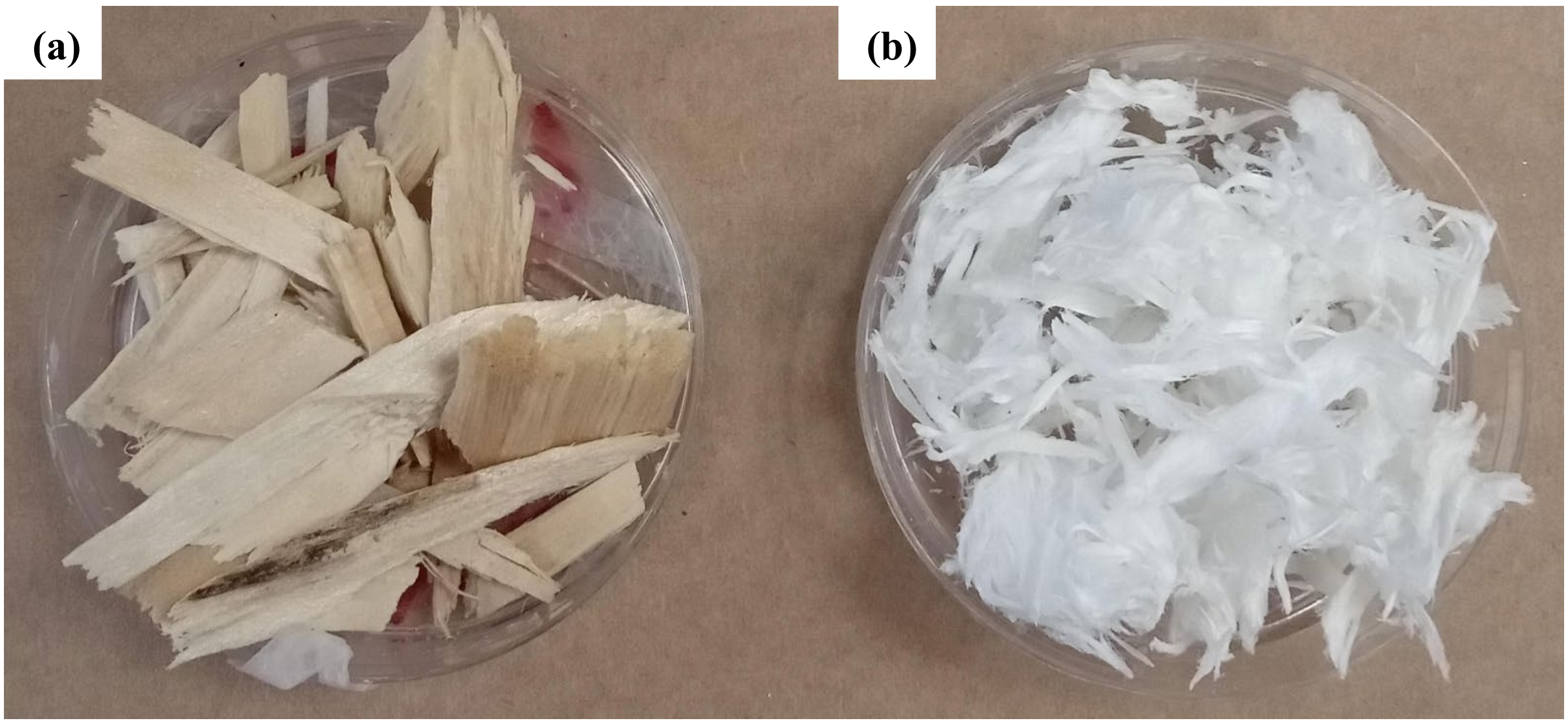

Figure S1. Digital image of (a) waste wood chips, and (b) white-coloured cellulose extracted from the same feedstock.

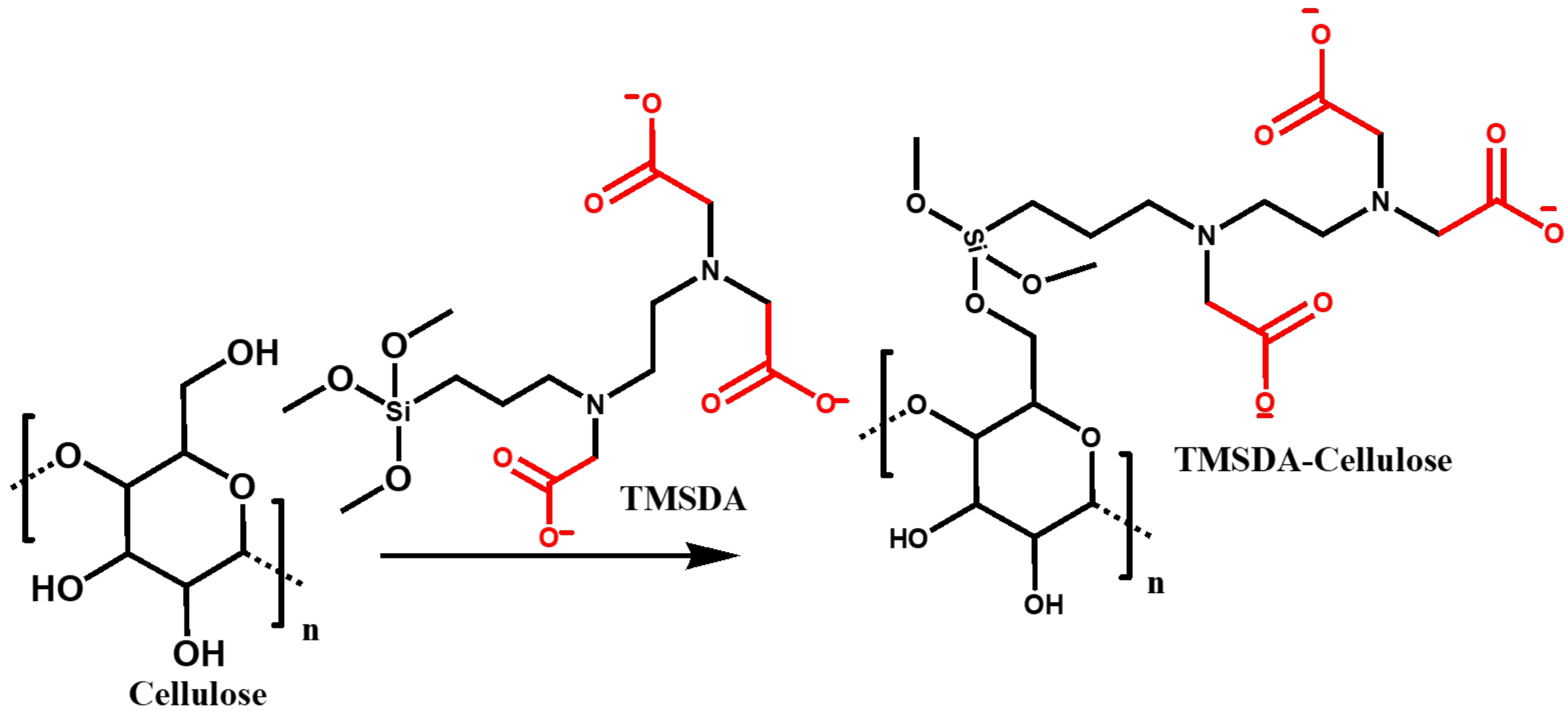

Scheme S1. Proposed TMSDA silanisation of cellulose: condensation at surface hydroxyl groups introduces tri-carboxylate functionality, yielding TMSDA-functionalised cellulose.

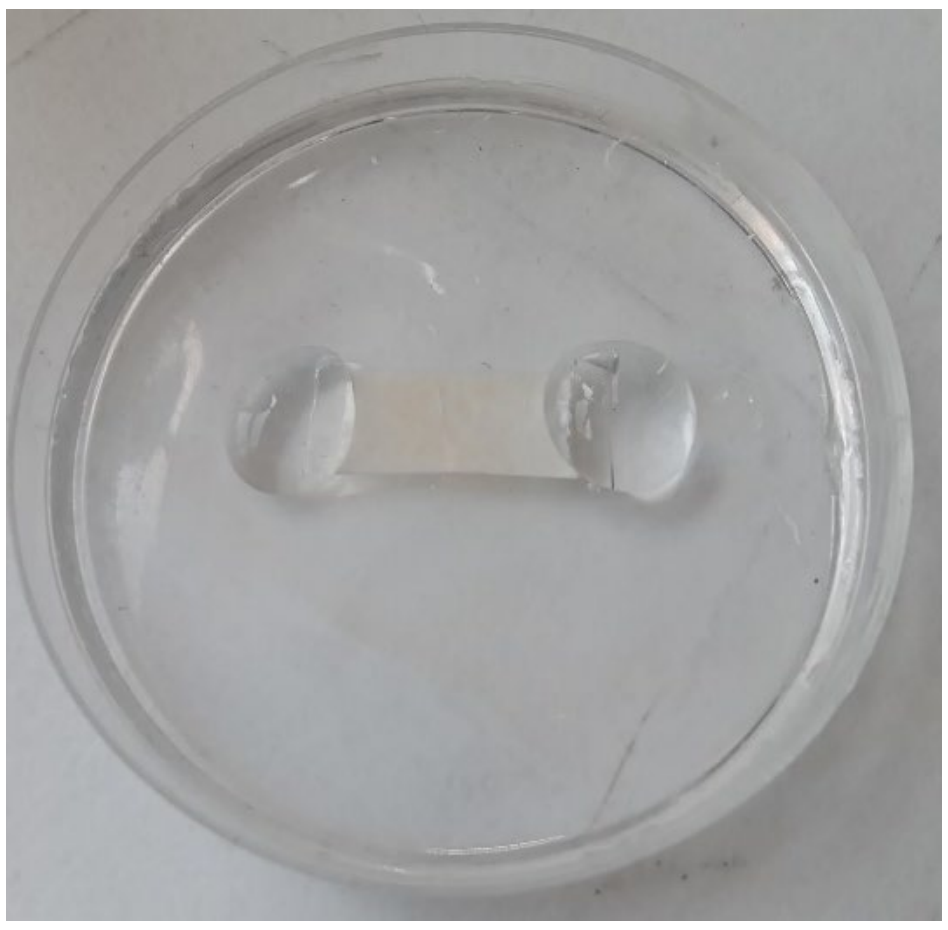

Figure S2. Membrane electrochemical test cell for ionic conductivity measurements. A cellulose membrane is embedded in SYLGARD-184 (PDMS) to prevent edge leakage; after curing, two isolated reservoirs are formed, exposing each membrane face to the electrolyte. Electrochemical measurements were performed using Ag/AgCl electrodes inserted into the reservoirs to monitor ion transport at applied potentials.

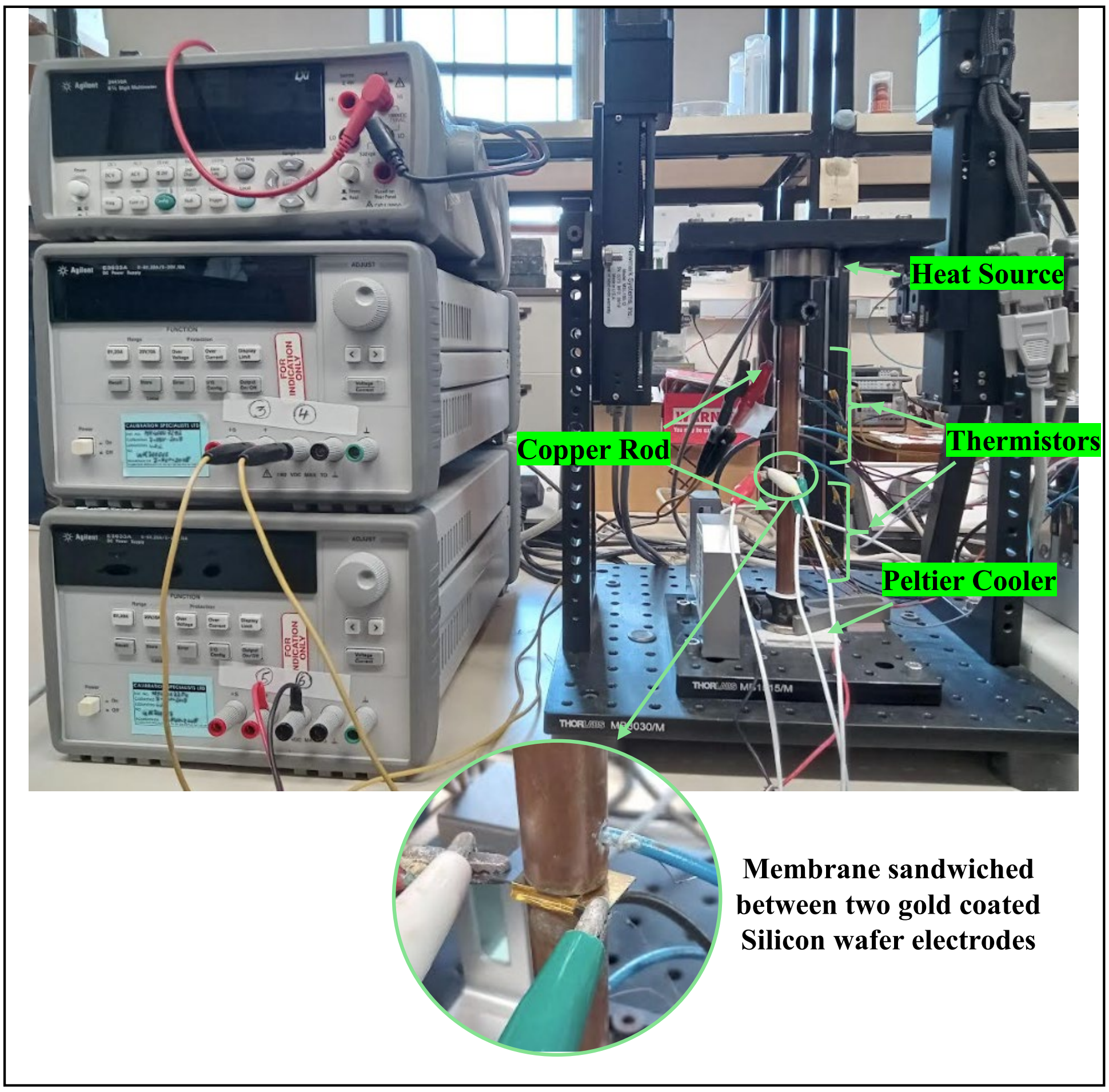

Figure S3. Modified ASTM 5470-06 based device setup to measure the cross-sectional ionic thermoelectric properties of stacked cellulose membranes. The apparatus consists of Agilent B2912A source-measure units used to control the temperature gradient and record electrical signals, integrated with a Thorlabs vertical test stage that enables precise positioning of heating and cooling blocks across the membrane sample. Electrical and thermal contacts are established using insulated leads and thermocouples. The close-up image shows the membrane positioned between two gold-coated silicon wafers, clamped between copper blocks, with thermocouple and voltage leads attached to measure local temperature and thermovoltage across the membrane.

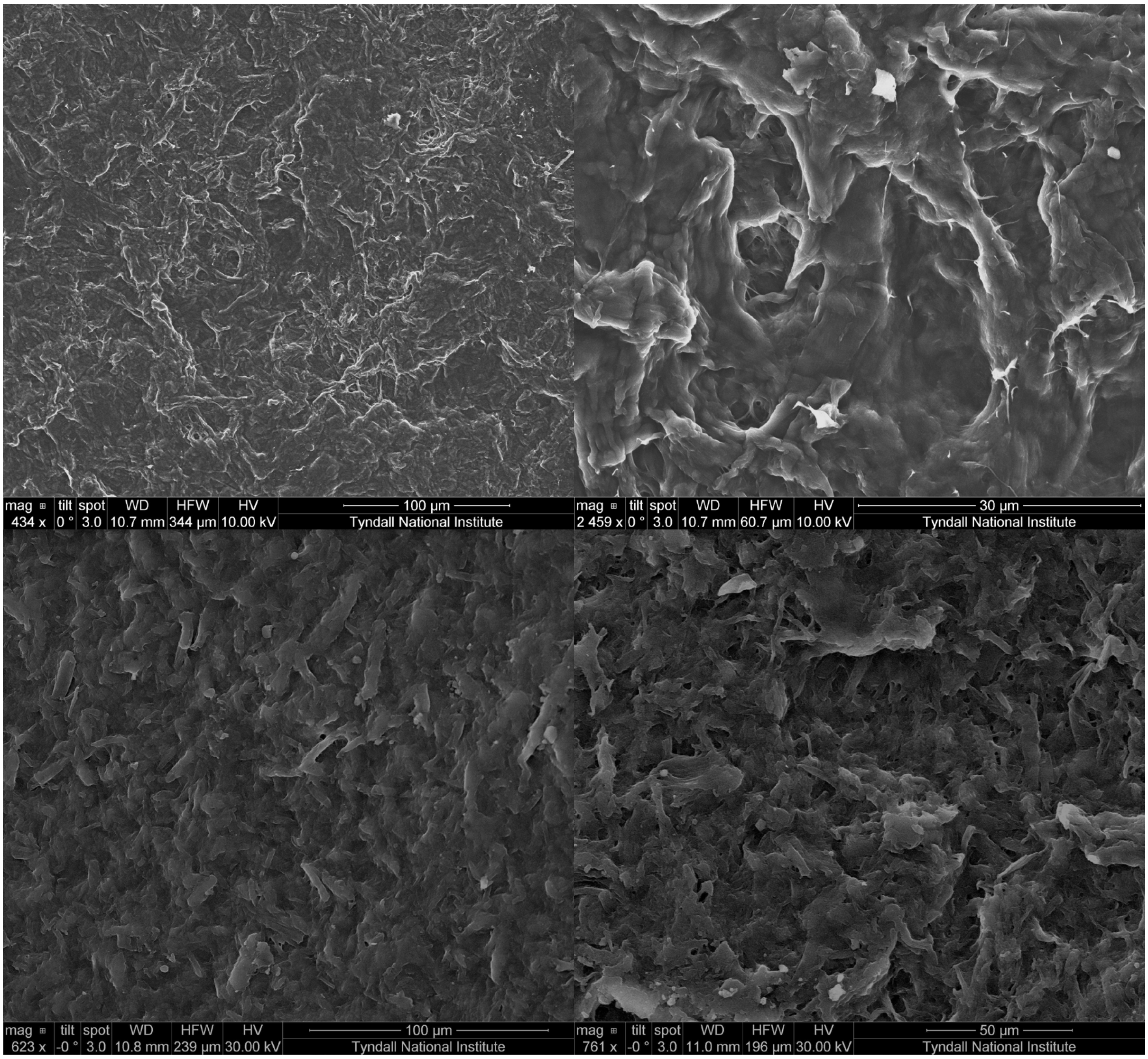

Figure S4. Scanning electron micrographs of the cellulose membrane surface: (a) lower magnification (scale bar, 100 µm & accelerating voltage 10 kV), (b) higher magnification (scale bar, 30 µm & accelerating voltage 10 kV), (c) lower magnification (scale bar, 100 µm & accelerating voltage 30 kV) and (d) higher magnification (scale bar, 50 µm & accelerating voltage 30 kV)

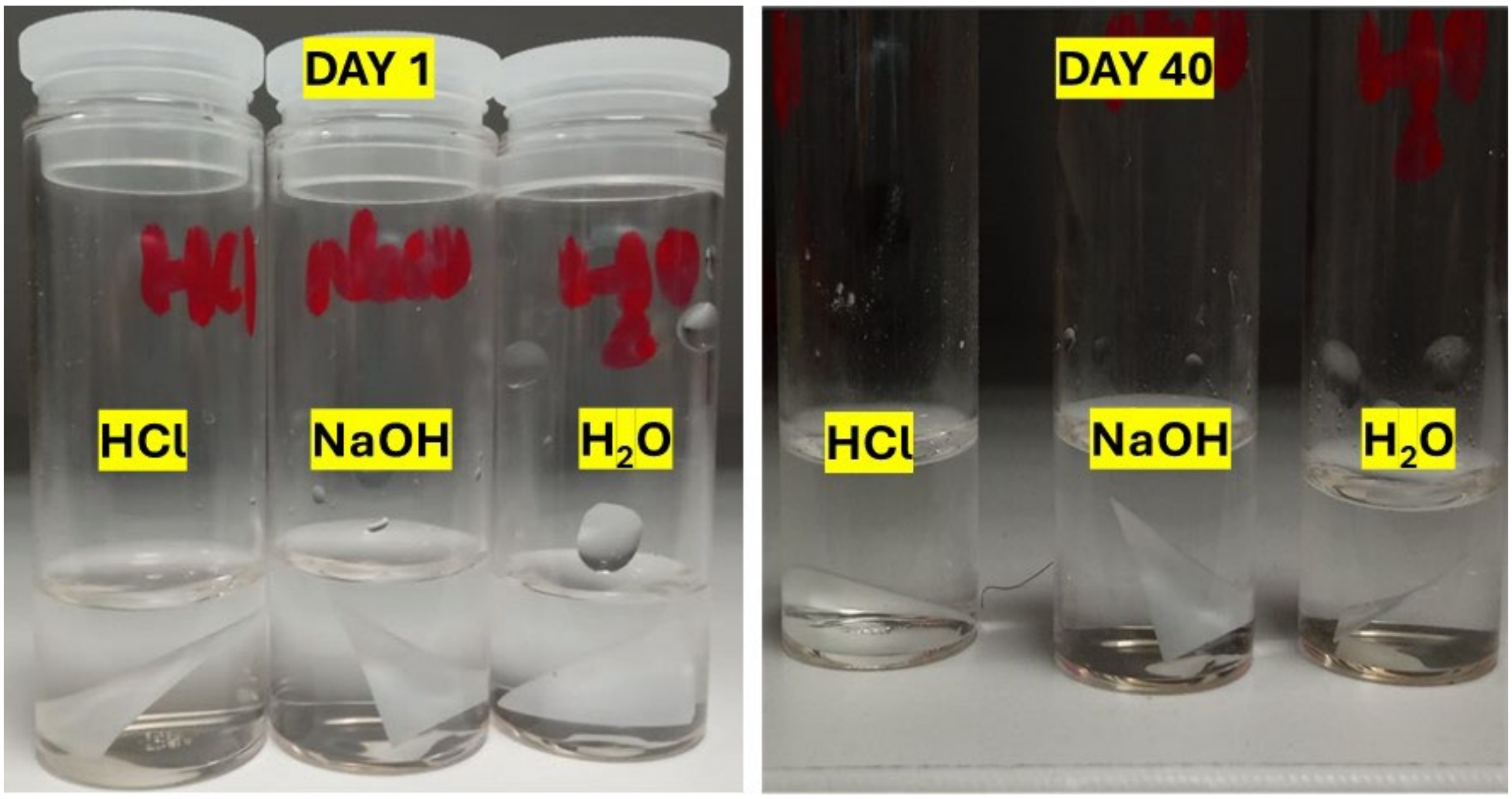

Figure S5. Macroscopic stability of lab-prepared cellulose membranes immersed in deionised water, 1 M NaOH and 1 M HCl. Photographs taken at Day 1 and Day 40 show no visible macroscopic degradation or delamination.

Table S1. LSV-derived mean ionic conductivity (κ) values for cellulose membranes (pristine, TEMPO-oxidised, and TMSDA-functionalised) compared with bulk KCl, used to construct Figure 4(c).

**[KCl] (M)**	κ_{bulk} **(mS cm^{-1})**	$\kappa_{pristine}$ **(mS cm^{-1})**	κ_{TEMPO} **(mS cm^{-1})**	κ_{TMSDA} **(mS cm^{-1})**
10^{-5}	0.00499	0.116	0.29	0.745
10^{-4}	0.01725	0.121	0.45	1.51
10^{-3}	0.1209	0.15	0.6	2.7
10^{-2}	1.439	0.211	1.98	8.44
10^{-1}	13.2	0.7	10.93	101.04
1	112.8	6.86	98.2	293.75

Values in Table S1 are reported as mean LSV-derived conductivities from three independently measured membrane devices (n = 3) and are shown without uncertainties for clarity. Across the dilute-to-intermediate concentration range 10^{-5}-10^{-2} M, the conductivity trends were reproducible, with TMSDA-functionalised cellulose consistently exhibiting higher κ than TEMPO-oxidised and pristine cellulose. Values at 0.1 and 1 M KCl are reported as apparent membrane-in-device conductivities calculated using the macroscopic device geometry. These high ionic-strength values were not used to extract intrinsic surface transport parameters because the measured resistance in this regime can be strongly influenced by wetting, contact resistance, effective hydrated transport geometry, and device-level conduction pathways. Consequently, greater device-to-device variability is expected at 0.1 and 1 M than in the dilute-to-intermediate concentration range used for surface–bulk transport analysis.

Table S2. Fitted parameters for bulk KCl, pristine cellulose, TEMPO-oxidised cellulose and TMSDA-functionalised cellulose from the conductivity models described in Equations 3, 4 and 6 of the main text. The membrane data were fitted over 10^{-5}-10^{-2} M KCl, corresponding to the dilute-to-intermediate concentration range used for surface-bulk transport analysis. High-ionic-strength points at 0.1 and 1 M were excluded from parameter extraction. Dashes denote parameters not applicable to a given model.

System	κ_{surf} (mS cm^{-1})	μ_{eff} (mS cm^{-1} M^{-1})	c^* (M) / × 10^{-2}	Λ^0 (S cm^2 mol^{-1})	K (S cm^2 mol^{-1} M$^{-1/2}$)	Fit range (M)
**Bulk KCl**	-	-	-	140.99	28.19	10^{-5}- 10^{-2}
**Pristine cellulose**	0.134	6.85	4.34	-	-	10^{-5}- 10^{-2}
**TEMPO-Cellulose**	0.67	98	0.50	-	-	10^{-5}- 10^{-2}
**TMSDA-Cellulose**	1.26	1.01×10^3	0.167	-	-	10^{-5}- 10^{-2}

Here, μ_{eff} is an empirical concentration-scaling coefficient for the bulk-like conductivity contribution within the electrolyte-filled pore network. It is not a true ionic mobility.

EIS fitting procedure and parameters

Electrochemical impedance spectroscopy (EIS) measurements were carried out over the frequency range 10^6-10^2 Hz with an AC perturbation amplitude of 10 mV (rms). Nyquist plots for KCl concentrations from 10^{-5} to 10^{-2} M are shown in Figure S6. Over this concentration range, the spectra show a single semicircle and were fitted using an equivalent circuit consisting of a series resistance (R_1) in series with a parallel ($R_2 \parallel C_2$) element (EC-Lab software). The corresponding impedance is given by Equation S1:

$$Z(\omega) = R_1 + \left(\frac{1}{R_2} + j\omega C_2\right)^{-1}$$

where (R_1) is the series resistance associated with the electrolyte, electrodes and external connections, (R_2) is the effective membrane/device resistance, (C_2) is the effective interfacial/membrane capacitance, (j) is the imaginary unit and ω is the angular frequency. Initial guesses were taken from the high- and low-frequency intercepts on the real axis, and the fits were iterated until the change in parameters between successive iterations was below 1 %. The fitted values of R_1, R_2 and C_2 for the cellulose paper membranes are given in Table S3

Table S3. Equivalent-circuit parameters (R_1, R_2, C_2) extracted from EIS spectra of TMSDA-cellulose membranes in KCl solutions (10^{-5} – 10^{-2} M).

[KCl] (M)	R_1 (Ω)	R_2 (Ω)	C_2 (pF)
10^{-5}	932	3.13×10^5	18.2
10^{-4}	957	1.93×10^5	18.8
10^{-3}	672	1.21×10^5	19.2
10^{-2}	900	4.08×10^4	22.1

The characteristic frequency f_0 associated with the semicircle is given by $f_0 = 1/2\pi R_2 C_2$ and lies between approximately 10^4 and 10^5 Hz for these concentrations, within the measured frequency window. The near-constant effective capacitance and monotonic decrease in (R_2) with increasing KCl concentration are consistent with a similar wetted device geometry across the concentration series and an increasing contribution from ionic conduction through the membrane pores.

At 1 M KCl, the impedance spectra show only a partial, highly compressed arc within the accessible frequency range. Fits using several plausible equivalent-circuit models gave comparable but poorly constrained parameters; therefore, the 1 M EIS data were not used for quantitative conductivity extraction. Reliable impedance spectra could not be obtained at 0.1 M KCl under the present measurement conditions. For consistency with the main text, the 0.1-1 M conductivities are therefore reported from LSV and interpreted as apparent membrane-in-device conductivities under the geometrical normalisation of Equation 2 in the main text.

(a) 10^{-5} M KCl

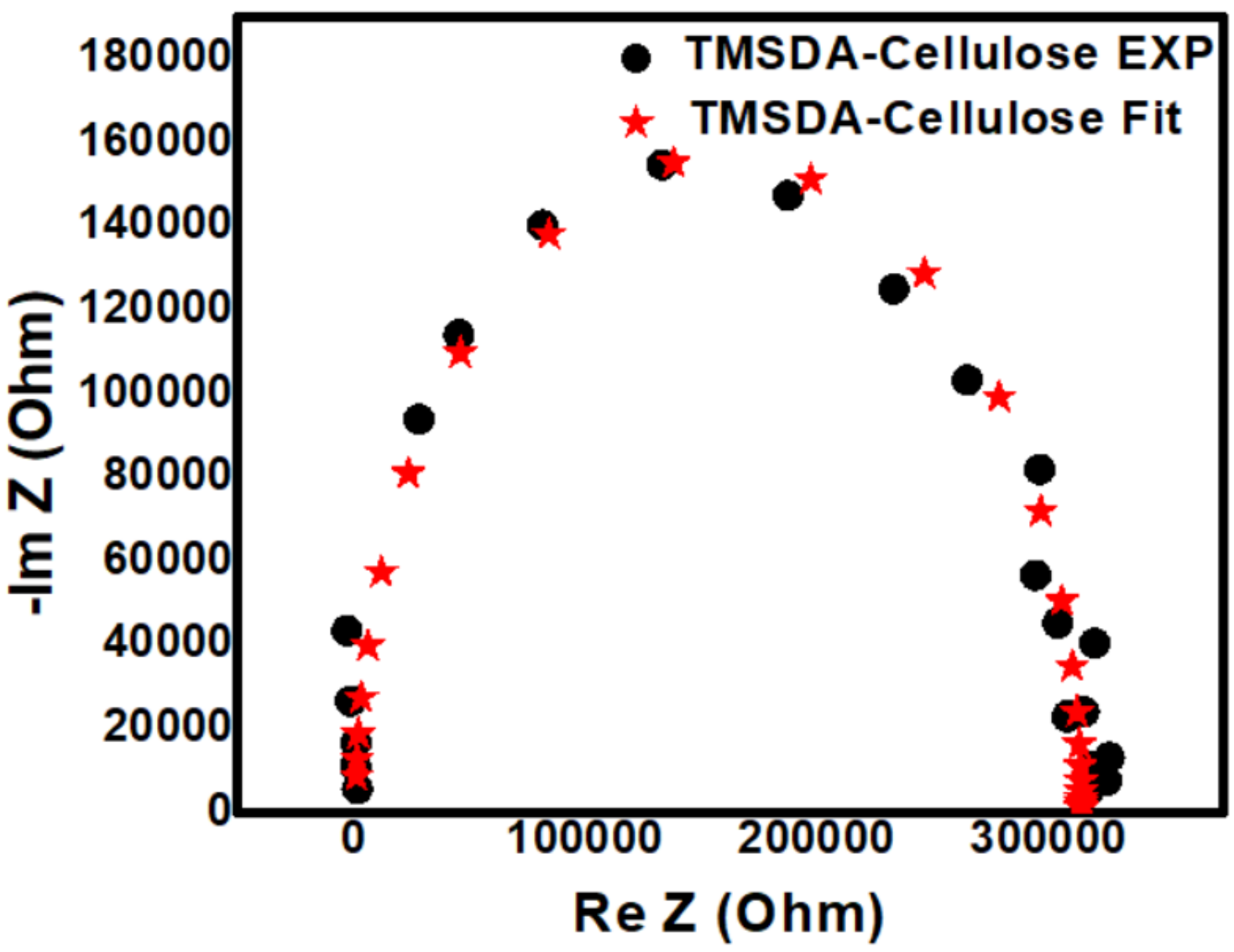

(b) 10^{-3} M KCl

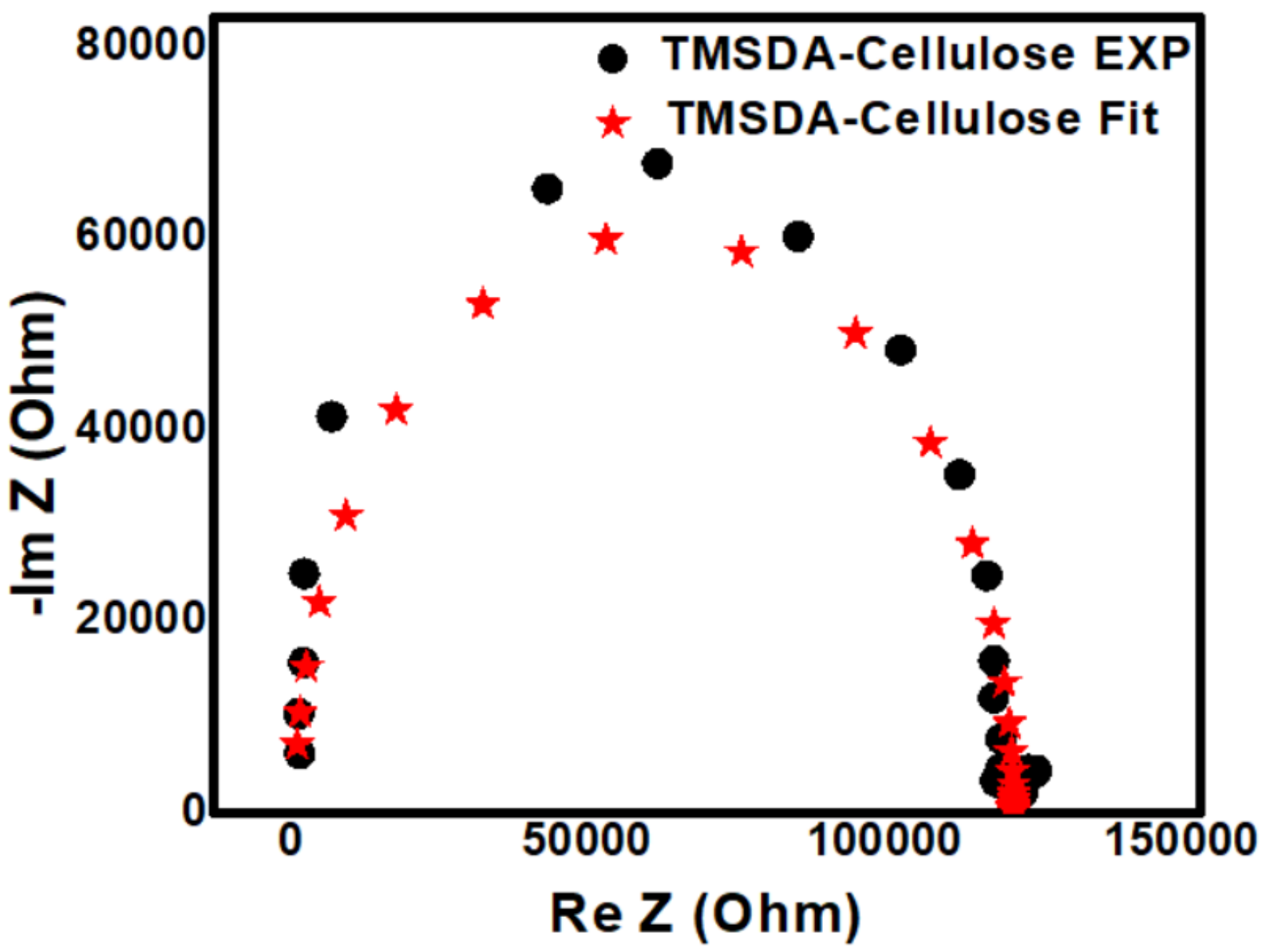

Figure S6. Representative Nyquist plots and equivalent-circuit fits for TMSDA-functionalised cellulose membranes infiltrated with (a) 10^{-5} M and (b) 10^{-3} M KCl. Symbols represent experimental data and solid lines represent fits to the circuit $R_1 + (R_2 \parallel C_2)$.

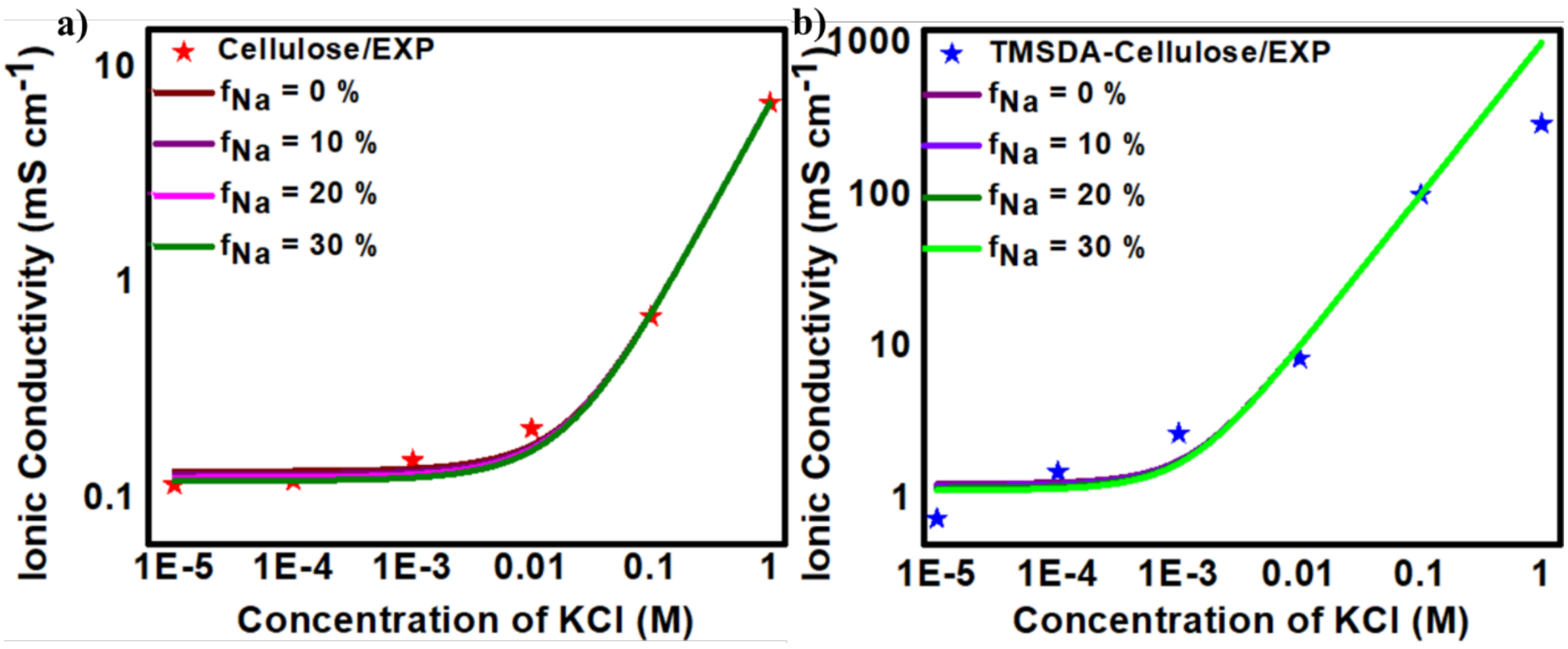

Figure S7. Modelled sensitivity of the fitted conductivity response to possible residual Na^+ counter-ions in (a) pristine cellulose and (b) TMSDA-functionalised cellulose membranes. Symbols show experimental KCl conductivity data. Curves show model fits in which κ_{surf} is rescaled according to the assumed fraction of residual Na^+ counter-ions using the mobility-weighted correction described in Equations 9 and 10 of the main text. This analysis assesses the sensitivity of the fitted parameters to possible Na^+ carryover and does not represent a direct measurement of residual Na^+ content.

Table S4. Benchmarking of apparent surface-conductivity parameters for the present cellulose membranes against representative cellulose-based and nanofluidic membranes reported in the literature. W here available, the apparent surface conductivity or characteristic low-concentration conductivity and crossover concentration (c^*) are reported.

Membrane system	Electrolyte	Reported Conductivity Parameter (mS cm^{-1})	c^* (M)	Reference
**Pristine waste-wood cellulose membrane**	KCl	0.134	4.34×10^{-2}	[This Work]
**TMSDA-functionalised waste-wood cellulose membrane**	KCl	1.26	1.67×10^{-3}	[This work]
**Commercially available regenerated cellulose 200 nm (RC) pore size membrane with quaternary amino group attached (CHMAC-RC-200)**	HCl	0.97	1.53×10^{-3}	[1]
**TEMPO oxidised RC-200 membranes**	HCl	0.46	3.07×10^{-3}	[1]
**Aligned cellulose nanofibre membrane**	KCl	1.1	-	[2]
**Carboxylated aligned cellulose nanofibre membrane**	KCl	2	-	[2]
**Aligned bacterial cellulose functionalised with quaternary ammonium groups**	KCl	1.2	-	[3]
**TEMPO-oxidised bacterial cellulose**	Under humidity	0.35	-	[4]
**Cellulose modified with conductive metal organic framework (Ni-based)**	NaCl	10^{-5} - 1 based on pH	$\leq 10^{-3}$	[5]

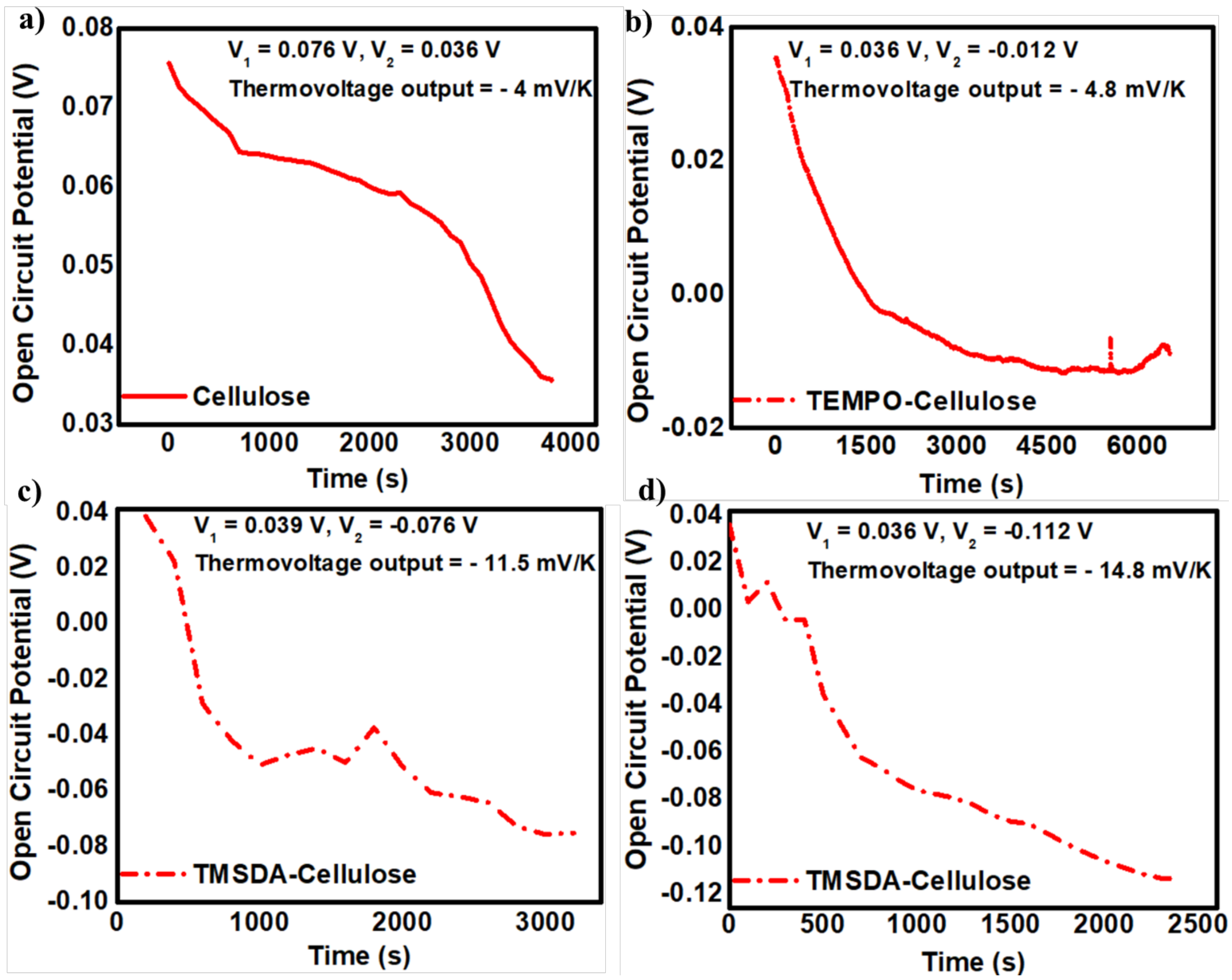

Figure S8. Open-circuit voltage, V_{oc}, versus time for cellulose membrane stacks infiltrated with 10^{-4} M KCl and measured in a sandwich cell with gold-coated Si electrodes under an applied temperature difference of 10 K: (a) pristine cellulose, (b) TEMPO-cellulose and (c, d) TMSDA-functionalised cellulose replicate measurements. Annotated values correspond to apparent single-point ionic Seebeck coefficients calculated as ($S_i = V_{oc}/\Delta T$). The sign of S_i follows the measured voltage polarity between the hot and cold electrodes. Each stack contained five membranes.

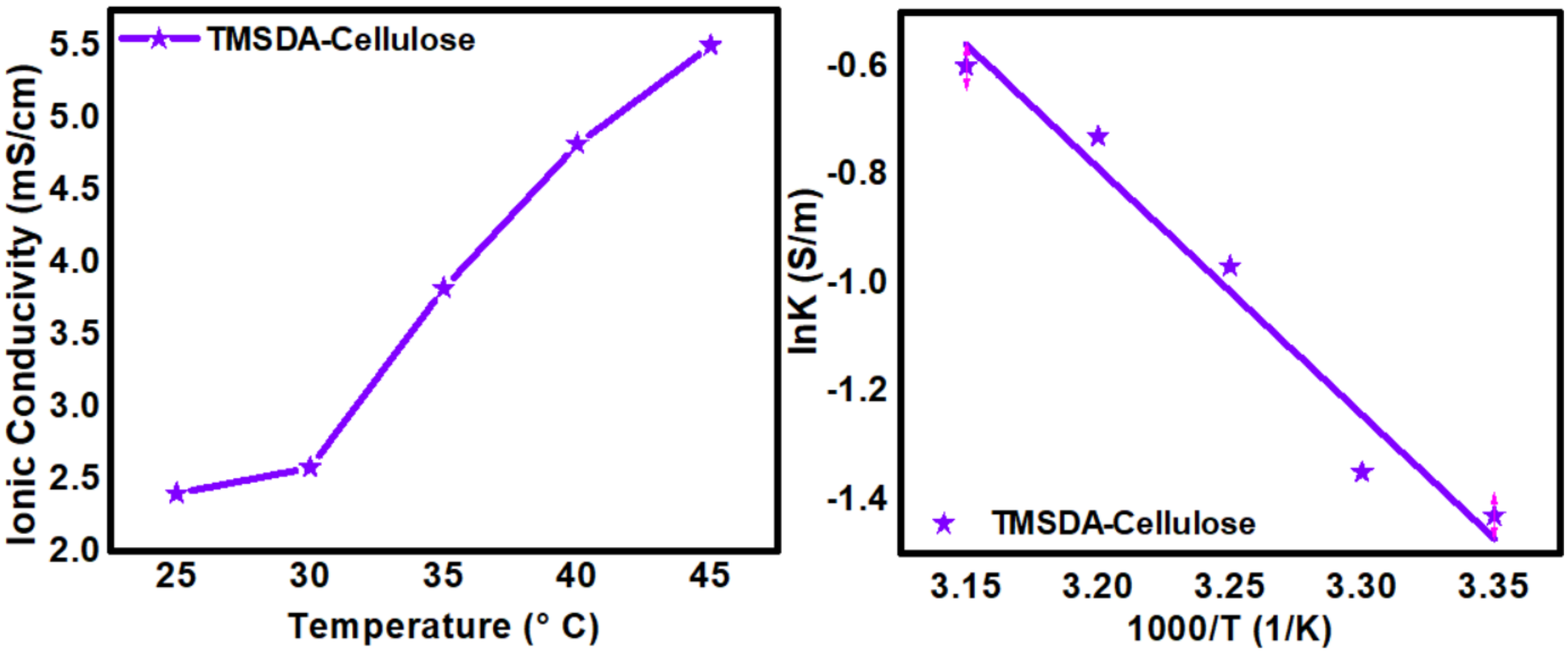

Figure S9. (a) Temperature-dependent ionic conductivity, κ, of a TMSDA-functionalised cellulose device infiltrated with 10^{-4} M KCl. (b) Arrhenius plot of lnκ versus (1000/T); the solid line shows the linear fit used to extract the apparent activation energy for ion transport. The fit gives E_a = 0.37 eV over the measured temperature range.

References

1. A. Ashokan, K. Rahme, R. R. Palanisamy, N. Padmanathan, K. M. Razeeb, S. Biswas and J. D. Holmes, *J. Mater. Chem. A*, 2025, **13**, 33671-33684.
2. T. Li, S. X. Li, W. Kong, C. Chen, E. Hitz, C. Jia, J. Dai, X. Zhang, R. Briber, Z. Siwy, M. Reed and L. Hu, *Sci. Adv.*, 2019, **5**, eaau4238.
3. Z. Wu, P. Ji, B. Wang, N. Sheng, M. Zhang, S. Chen, and H. Wang, *Nano Energy*, 2021, **80**, 105554.
4. D. Rahmadiawan, H. Abral, M. A. Azka, S. M. Sapuan, R. I. Admi, S. C. Shi, R. Zainul, A. Zikri and M. Mahardika, *RSC Adv.* 2024, **14**, 29624-29635.
5. S. Zhou, Z. Qiu, M. Strømme, and C. Xu, *Energy Environ. Sci.* 2021, **14**, 900-905.
